# Evolution of the Knowledge of Free Radicals and Other Oxidants

**DOI:** 10.1155/2020/9829176

**Published:** 2020-04-23

**Authors:** Sergio Di Meo, Paola Venditti

**Affiliations:** Università degli Studi di Napoli Federico II Dipartimento di Biologia, Complesso, Universitario Monte Sant'Angelo, Via Cinthia, I-80126 Napoli, Italy

## Abstract

Free radicals are chemical species (atoms, molecules, or ions) containing one or more unpaired electrons in their external orbitals and generally display a remarkable reactivity. The evidence of their existence was obtained only at the beginning of the 20th century. Chemists gradually ascertained the involvement of free radicals in organic reactions and, in the middle of the 20th century, their production in biological systems. For several decades, free radicals were thought to cause exclusively damaging effects . This idea was mainly supported by the finding that oxygen free radicals readily react with all biological macromolecules inducing their oxidative modification and loss of function. Moreover, evidence was obtained that when, in the living organism, free radicals are not neutralized by systems of biochemical defences, many pathological conditions develop. However, after some time, it became clear that the living systems not only had adapted to the coexistence with free radicals but also developed methods to turn these toxic substances to their advantage by using them in critical physiological processes. Therefore, free radicals play a dual role in living systems: they are toxic by-products of aerobic metabolism, causing oxidative damage and tissue dysfunction, and serve as molecular signals activating beneficial stress responses. This discovery also changed the way we consider antioxidants. Their use is usually regarded as helpful to counteract the damaging effects of free radicals but sometimes is harmful as it can block adaptive responses induced by low levels of radicals.

## 1. Introduction

The term “radical” was first introduced by Guyton de Morveau in 1786 and later used by Gay-Lussac, Liebig, and Berzelius to indicate groups of atoms which were found unchanged in many substances (see Solov'ev [1]). The introduction of the term is generally attributed to Liebig and Wöhler, who in 1832 published a paper reporting that, in the various transformations of the essence of bitter almonds (the benzoic aldehyde) and of its derivatives containing chlorine and bromine, the radical, to which the formula C_14_H_10_O_2_ was attributed, remained unchanged [2]. The work of Liebig and Wöhler exercised a considerable influence on the development of organic chemistry. After the classification of organic compounds in homologous series, the radicals were considered as groups of atoms, belonging to molecules of organic compounds, which are kept unaltered in reactions involving the functional group of the compounds and which can be replaced by other radicals without modifying these reactions substantially.

Despite the interest in the radicals and their chemistry, their isolation was considered impossible, and, until the end of the nineteenth century, no direct evidence of their independent existence was found. In 1840, Berzelius believed that the inability to isolate the radicals did not depend on the fact that they did not exist but on the fact that they were combined too quickly and that the methods available in that time were insufficient for their isolation (see Solov'ev [1]).

Presumably, the first reaction involving free radicals was that of Fenton in 1894 [3]. He noted that when to a small amount of a tartaric acid solution a drop of diluted solution of ferrous sulphate was added, followed by a drop of hydrogen peroxide and finally an excess of caustic alkali, a light violet colour was obtained. The observed change was proposed as a distinguishing test for tartaric acid. Free radicals were not known at that time, and only three decades later, the involvement of the hydroxyl radical (^·^OH) was proposed by Haber and Wilstätter [4]. Currently, oxidation processes that use H_2_O_2_ activation by iron salts, classically referred to as Fenton's reagent, are known to be very effective in destroying many dangerous organic pollutants in water. Furthermore, the Fenton reaction plays a very important role in free radical biology and medicine.

However, the path taken to recognize the possibility of the independent existence of free radicals and their fundamental importance for living systems has been long and not without obstacles. This review is aimed at retracing this path by highlighting the steps that have made a fundamental contribution to understanding the role played by free radicals in biological systems (Table 1).

## 2. Free Radical Isolation

Only at the beginning of the last century, evidence was found that isolation of organic free radicals with a measurable lifetime was possible following the preparation of the triphenylmethyl radical, (C_6_H_5_)_3_C^·^, carried out by Gomberg [5].

In this compound, obtained during the attempt to synthesize the hydrocarbon hexaphenylethane, (C_6_H_5_)_3_-C-C-(C_6_H_5_)_3_, the central carbon is trivalent since it is combined with three substituents instead of four and presents an unshared electron. Free radicals of the triphenylmethyl type are stable only in certain organic solvents; they are rapidly destroyed by irreversible reactions in the presence of air, water, or strong acids.

A whole range of other aryl-substituted analogous compounds was prepared soon afterwards, and Gomberg's inference that the hexaphenylethane dissociated into two free radicals was apparently substantiated by carrying out molecular weight determinations [6, 7].

It is interesting that this discovery involved stable free radicals. Generally, most free radicals are extremely reactive and, consequently, short-lived species. The great chemical reactivity of the free radicals is to be associated with the available combining energy of the odd electron and their reactions, whenever possible, resulting in the completion of electron pairs. The reason for the relative stability of triphenylmethyl and its analogues, which favoured their isolation, was not an easy problem to solve for theoretical chemists. However, the application of wave mechanics to organic chemistry led to an extended conception of *resonance* within complicated molecules, and it was realized that the domain of the odd electron of triphenylmethyl, like that of the aromatic sextet of benzene, may extend over a large region of intramolecular space. In consequence, much less intrinsic energy is associated with the free valence electron in the complex molecule of the triphenylmethyl than in more simple compounds. However, a relatively stable species, such as triphenylmethyl, is not commonly found, and the technology in that year could not handle transient entities with a very short life so that the research on free radicals had to await further developments.

In many quarters, Moses Gomberg's announcement of the triaryl methyl type free radicals was greeted, even as late as 1930, with disbelief or, at least, disinterest. Still, in 1915, von Richter wrote: “The assumption of the existence of free radicals, capable of existing alone and playing a special role in chemical reactions, has long been abandoned” [8]. A decade later Porter, at Berkeley, said: “Negative results gradually established the doctrine that a free carbon radical was incapable of independent existence” [9], even though the evidence of the existence of free radicals continued to increase.

Although measurements of the vapour densities of gases at high temperatures had indicated that diatomic molecules could dissociate into free atoms, the possibility of the independent existence, at normal temperatures, of free atoms such as hydrogen, oxygen, or chlorine received almost no consideration until 1913, when Bohr showed that the spectrum emitted from a hydrogen discharge tube could be interpreted as an emission spectrum of an atomic form and not of a molecular form of hydrogen [10].

In 1922, Wood [11] first isolated and described the properties of atomic hydrogen, which he produced in an electrical discharge tube. The chemical properties of atomic hydrogen were investigated more fully by Bonhoeffer in 1924 [12].

Subsequently, Paneth and Hofeditz [13] prepared the free radical methyl (^·^CH_3_), by pyrolysis of tetramethyl lead using an adaptation of the system used by Bonhoeffer to study atomic hydrogen.

In the subsequent years, the extensive and careful work of Gomberg and other authors who carried on in his field, together with the work on gas-phase free radicals initiated by Paneth, led to a body of experimental evidence for stable as well as short-lived free radicals, so that free radicals gained respectable status in chemical circles.

## 3. Free Radical in Chemical Reactions

Considering the significant progress made in the field of free radical isolation, it is not surprising that chemists would start to introduce free radical hypotheses into their reaction mechanisms. In 1933, Kharasch and Mayo [14] invoked a free radical mechanism for the addition of hydrogen bromide to olefins which was subsequently applied to other chemical systems.

Before 1933, the orientation of the addition of hydrogen bromide to alkenes was the subject of much confusion. Sometimes, the addition took place according to Markovnikow's rule [15], which established that the acidic proton adds to the less substituted carbon of the double bond. This is because the carbocation obtained from this addition is stabilized by the presence of electron repellent alkyl group(s):

(1) $$\begin{array}{c} {\text{CH}}_{3}-\text{CH}={\text{CH}}_{2}+{\text{H}}^{+}⟶{\text{CH}}_{3}-\overset{+}{\text{C}}-{\text{CH}}_{3}\overset{{\text{Br}}^{-}}{⟶}{\text{CH}}_{3}-\text{CHBr}-{\text{CH}}_{3} \end{array}$$

However, at other times, addition took place in the opposite way as the hydrogen added to the most substituted carbon. The mystery was solved in 1933 by the research of Kharasch and Mayo who explained how an anti-Markovnikow orientation could be achieved via free radical addition [14]. The factors able to explain this process turned out to be organic peroxides present in the alkenes-peroxides that were formed by the action of atmospheric oxygen on the alkenes. Indeed, Kharasch and Mayo found that when alkenes that contained peroxides or hydrogen peroxide reacted with hydrogen bromide, anti-Markownikoff addition of hydrogen bromide occurred. Thus, they proposed that the anti-Markownikoff addition of HBr was due to the presence of peroxides and termed this the “peroxide effect,” which was thought to proceed through a free radical chain addition reaction in which the intermediate carbon-centred radical was stabilized by the adjacent alkyl group(s):

(2) $$\begin{array}{c} \text{R}-\text{O}-\text{O}-\text{R}⟶2\text{R}-{\text{O}}^{\cdot }\overset{2\text{HBr}}{⟶}2\text{R}-\text{OH}+2{\text{Br}}^{\cdot }\\ {\text{CH}}_{3}-\text{CH}={\text{CH}}_{2}+{\text{Br}}^{\cdot }⟶{\text{CH}}_{3}-\overset{•}{\text{C}}\text{H}-{\text{CH}}_{2}-\text{Br}\overset{\text{HBr}}{⟶}{\text{CH}}_{3}-{\text{CH}}_{2}-{\text{CH}}_{2}‐\text{Br}+{\text{Br}}^{\cdot } \end{array}$$

Kharasch also suggested that radical intermediates and chain reactions can play an important role in many organic reactions, and over the following years, he succeeded in developing synthesis reactions, including sulfonation [16], chlorination [17], and carboxylation [18] of hydrocarbons and paraffin.

His work paved the way to the development of synthetic materials ranging from rubber to plastics. Conventional polymerization for condensation continued to be used to produce nylon and other products. But free radical polymerization had advantages such as high tolerance of chemical impurities and extreme temperatures and the ability to be used with a wide range of monomers (organic molecules). Today, free radicals are used to produce nearly half the polymers we use—materials used in everything from food wrapping to paint, adhesives, film, carpeting, piping, and more.

In this context, the work of Semenov, whose researches fall into the field of chemical kinetics and the processes of combustion, is significant [19]. The outcome of these researches is the certainty that many chemical reactions are aided by free radicals which are produced during the process. Semenov's work also opened a new path for understanding the connection between the reactivity and the structure of particles entering a chemical reaction and created the possibility of rationally regulating the rate and direction of chemical changes. This, in turn, had profound consequences for the improvement of consolidated industrial processes and for the development of new processes, for example, in the fields of polymerization and direct oxidation as well as hydrocarbon cracking. Interestingly, some concepts of Semenov concerning chain reactions were also formulated by the renowned English kinetic chemist C. N. Hinshelwood [20], who was awarded the Nobel Prize in Chemistry jointly with Semenov in 1956 (Table 2).

It is worth noting that antioxidants were used for the first time in the nineteenth century in the rubber industry when it was observed that some molecules, identified empirically, could slow the degradation and allow optimization of the process of vulcanization. Moureau and Dufraisse [21] were the first to propose an inclusive theory which should account for the effectiveness of almost infinitesimally small amounts of material in retarding or accelerating the processes of oxidation.

It has also been suggested [22] that Cummings and Mattill [23], who in 1931 realized the idea of the function of vitamin E as an *in vivo* lipid antioxidant, were perhaps inspired by the work on autoxidation by Moureau and Dufraisse [21].

In terms of antioxidants, a milestone in the evolution of knowledge was the work of Albert Szent-Györgyi, who, in the thirties, isolated a strongly reducing substance that he called “hexuronic acid” [24], and that was subsequently identified as vitamin C [25].

The same researcher and his collaborators studied the antioxidant effect of polyphenols found in plants governing vascular permeability and proposed the name vitamin P [26] but could not withstand the criticism of the absence of deficiency syndrome, as fundamental to the definition of a function vitamin.

A further significant contribution to the free radical chemistry was the concept found in Haber's final paper [27] that the highly reactive hydroxyl radical could be generated from an interaction between superoxide (O_2_^·^) and hydrogen peroxide (H_2_O_2_):

(3) H2O2+O2·−⟶O2+O·H+OH−

The authors recognized that the reaction is thermodynamically unfavourable in biologic systems, having a second-order rate constant of zero in aqueous solution, and would require some sort of catalyst to proceed. Thus, they discussed the need for a metal ion catalyst and illustrated that the net reaction creating the hydroxyl radical can be broken down into two chemical reactions (Equations (4) and (5)).

The former predicts that hydrogen peroxide is reduced at the iron centre with the generation of the hydroxyl free radical. This reaction is commonly referred to as Fenton reaction although Fenton never wrote it:

(4) H2O2+Fe2+⟶Fe3++O·H+OH−

The second indicates that superoxide recycles oxidized iron to the ferrous form:

(5) $$\begin{array}{c} {{\text{O}}_{2}}^{\cdot -}+{\text{Fe}}^{3+}⟶{\text{Fe}}^{2+}+{\text{O}}_{2} \end{array}$$

It is interesting that, although a lot of research was done to determine the nature of the species involved in Fenton reaction, the nature of these species is still under discussion. In fact, the popular theory, due to Haber-Weiss, involving the formation of ^·^OH radicals, has been questioned by studies suggesting an alternative interpretation of the Fenton reaction including the formation of the ferryl ion as active oxidizing species [28].

Chemical studies involving free radicals were ongoing for many years before it was recognized that such reactive species are produced in biological systems. At that point, it was quickly realized that the Haber-Weiss reaction might represent the major mechanism by which the highly reactive hydroxyl radical is generated in biological systems.

## 4. Free Radicals in Biological Systems

It is widely believed that the prolific work of Leonor Michaelis was responsible for the interest in free radicals as naturally occurring biochemical intermediates in biological systems. In investigations started in the 30s, Michaelis observed that the curves of oxidation-reduction potentials, obtained by adding increasing amounts of an oxidant to hydroquinone, initially showed the loss of an electron and then, in a subsequent stage, the loss of another electron [29]. He believed that the loss of the first electron corresponded to the formation of the free semiquinone radical. The second electron was given by the semiquinone with the formation of the completely oxidized form, the quinone.

Since the intermediate radicals are very unstable and quickly lose the second electron, their concentration in the reaction medium is very low, so it is very difficult, or even impossible, to identify them. Therefore, it is not always possible to differentiate oxidation reactions, which proceed in one step and involve radicals, from those that proceed by transferring two electrons at a time, relying exclusively on the products that are formed. However, Michaelis enunciated the theory that all the oxidations of organic molecules, which involve the exchange of two electrons, proceed through two successive stages, the intermediate stage being constituted by a free radical [30].

We know today that this theory is inaccurate in that there are biochemical reactions in oxidative reactions with loss of two electrons, which occur in a single step and which do not involve free radicals. However, Michaelis's ideas gave rise to research that indicated that some intermediate reagent can sometimes be identified in enzymatic oxidation-reduction reactions of biological molecules, even though there was no evidence that the intermediate was a free radical.

However, starting in 1954, using sensitive methods for detecting free radicals, such as electronic spin resonance (ESR), which uses the paramagnetism of free radicals, some researchers showed that in some enzyme-substrate systems, it was possible to identify a paramagnetic intermediate compound. One of the areas in which the ESR was validly used was the study of enzymatic oxidation. In such reactions, an electron is first removed by an enzyme from the substrate and then transferred to a coenzyme. An important example is the formation of a semiquinone during the riboflavin oxide-reduction (vitamin B2), which constitutes the flavin coenzymes, flavin mononucleotide (FMN), and the flavin adenine dinucleotide, involved in many oxidation-reduction reactions [31].

Later, it was shown that free radicals were much more widespread in biological systems than previously assumed. Free radicals were found not only in the case of oxidation-reduction processes but also in many reactions of biological interest such as photochemical reactions, photosynthesis, and bioluminescence.

The path, which has led to the onset and acceptance by the scientific community of the idea that free radicals are continuously formed in the cell as collateral products of normal metabolic reactions, can be initiated by the discovery of the mechanisms underlying the effects of oxygen toxicity and ionizing radiation.

## 5. Effects of Radiation

Evidence is available that physical or chemical environmental perturbations, of which radiation emission is perhaps the main contributor, can induce pathological effects through free radical mechanisms.

In 1895, the German physicist Röentgen discovered “a new kind of ray” which could blacken photographic films enclosed in a light-tight box [32]. He named these rays as X-rays, which meant unknown rays. In 1896, radioactivity was discovered by Antoine Henri Becquerel [33]. In the same year, Pierre and Marie Curie discovered and isolated radium successfully [34].

The potential hazards of radiations or radioactive materials were not adequately acknowledged at the beginning of their discoveries, although there was evidence that radiation produces biological damage. In fact, the first cases of human injury were reported in the literature just a few months following Röentgen's original paper announcing the discovery of X-rays (see Sansare et al. [35] for a review). Furthermore, as early as 1904, the first case of death occurred due to metastatic carcinoma induced by X-ray exposure.

Early human evidence of harmful effects because of exposure to radiation in large amounts existed in the 1920s and 30s, based upon the experience of early radiologists, miners exposed to airborne radioactivity underground, persons working in the radium industry, and other special occupational groups. However, the long-term biological significance of smaller, repeated doses of radiation, however, was not widely appreciated until relatively recently, and most of our knowledge of the biological effects of radiation has been accumulated since World War II. In fact, much of what we know today about the relationship between ionizing and cancer stems from studies conducted on people who survived the atomic bombs of Hiroshima and Nagasaki. Since these were very particular exposure conditions, it was difficult for a long time to understand whether those conclusions could be applied even at the most common levels of exposure. In recent years, several studies have confirmed that even low levels of exposure can give rise to the transformation of cells that leads to the development of cancer. The quantification of this risk, however, is very complex: it depends on several factors, including the dose to which one is exposed and the duration of exposure, the type of radiation, the areas of the body irradiated, and the age at which one has entered in contact with radiation.

In the first part of the last century, much progress was also made in understanding the effects induced by ionizing radiation on individual components of biological systems and the basic mechanism underlying such effects.

Early studies concerning the action of radiation on enzymes had led to the conclusion that X- and *γ*-rays only influence enzymes when the dose is enormous [36]. However, in 1940, Dale found that the reason for these failures was the use of large amounts of enzyme and of impure preparations [37]. Indeed, by decreasing the concentration of the enzyme, the carboxypeptidase, he obtained inhibition with relatively low doses of X-rays. Subsequent studies showed that when dilute solutions of enzymes were irradiated with X-rays, the enzymes requiring -SH groups for enzymatic activity were more susceptible to inhibition than enzymes that did not require –SH groups for activity [38]. Furthermore, the enzymes can be reactivated by the addition of glutathione. Thus, it was suggested that inhibition of sulfhydryl enzymes was due to oxidation of sulfhydryl groups, whereas inhibition of nonsulfhydryl enzymes, which required larger amounts of X-rays, was attributed to protein denaturation. The mechanism of –SH groups was explained by following Weiss's suggestion [39], of the liberation of free radicals resulting from the interaction of X-rays with the water molecules. In fact, ionizing radiation can directly damage different biologically essential macromolecules, such as DNA, membrane lipids, and proteins, through ionizations that create sites of electron loss (radical cations), electron gain (radical anions), and excitations. However, the action of ionizing radiation can also be indirect involving the primary formation of free radicals, such as H^·^ and ^·^OH, with subsequent formation of H_2_O_2_, atomic oxygen, and HO_2_^·^, which react with the macromolecules.

This indirect action already suggested by Risse [40] in his “activated solvent” hypothesis, and later developed by Fricke [41], had been resumed by Dale, who had postulated that carboxypeptidase enzyme molecules were not directly affected by the ionizing radiation, but indirectly through collision with a labile product resulting from water ionization [31].

The significant consequences of various types of radiation-induced DNA damages show that DNA is the principal target for the biological effects of radiation.

In the early days, radiation-induced DNA damages were studied under two different conditions, irradiating the DNA molecule either directly or in a dilute aqueous solution. These studies allowed to establish that, as for proteins, ionizing radiation damages DNA either directly by deposition of energy in the DNA resulting in the ejection of electrons leading to the formation of a free radical (R) or indirectly by the ionization leading to the formation of hydroxyl radicals and indirectly by reactions with radicals produced by ionizing H_2_O molecules.

It was also hypothesized that the free radical-mediated covalent modifications leading to oxidative damage to critical biomolecules during and immediately following irradiation result in most, if not all, of the biological effects of ionizing radiation. The most compelling evidence in favour of this hypothesis came from observations that manipulations of antioxidants (i.e., thiols, hydroxyl radical scavengers, and hydroperoxide metabolizing enzyme systems) at the time of irradiation appeared to alter the reactions of free radicals (and reactive oxygen species) leading to alterations in oxidative damage as well as alterations in the biological effects of IR [38, 42, 43].

It is worth noting that the radiosensitivity of the tissues is strongly dependent on oxygen concentration, a phenomenon named oxygen effect. This effect was first demonstrated by Schwartz in 1909 [44]. Using X- and *γ*-rays, he showed that firm pressure applied to the skin during irradiation greatly reduced the subsequent reaction as compared to irradiation under normal conditions. Later, Jolly found reduced damage in the guinea pig and rat thymus irradiated when the blood supply was occluded by a ligature [45], and Mottram observed similar protection from radiation damage by occluding the blood supply of the rat's tail by ligature of the vascular connections [46]. These findings were later confirmed by other researchers who used different animal species and tissues [47].

For a long time, the oxygen fixation hypothesis, developed in the late 1950s, has been widely regarded as the most satisfactory explanation of the oxygen effect [48]. Central to this hypothesis was the belief that most DNA damage produced by X-rays can be repaired, but that repair is more difficult or impossible when caused by the product of a radical and an oxygen molecule, so that the amount of stable DNA damage and the extent of lethality from a given dose increase [48]. In fact, the actual role of oxygen in the inactivation mechanism represents still an open problem; in particular, it has been shown that oxygen fixation hypothesis cannot be regarded as maintainable more and, on the other hand, has argued that the oxygen effect can be hardly a simple consequence of greater reactivity of oxygen radicals [49]. Such an explanation of the oxygen effect is based on the possibility that the primary radicals, H^·^ and *e*_aq_^−^ react with O_2_, generating HO_2_^·^ and O_2_^·-^, respectively:

(6) $$\begin{array}{c} {\text{H}}^{\cdot }+{\text{O}}_{2}⟶{\text{HO}}_{2}^{\cdot }\\ e_{\text{aq}}^{-}+{\text{O}}_{2}⟶{\text{O}}_{2}^{\cdot -} \end{array}$$

This should lead to the generation of new free radicals which amplify the radiation effect. On the other hand, it is not possible to exclude that a free radical generated from a biological molecule, by direct effect or by extraction of one hydrogen by ^·^OH, can bind to O_2_ forming a peroxyl radical, which can fix the damage in a relatively unrepairable condition, and extract one hydrogen from another molecule causing a chain reaction.

It is also worth noting that, while classical radiation toxicity models identify DNA damage as the universal critical lesion in cells [50], studies now support that the survival of many organisms is governed by the level of oxidative protein damage caused during irradiation [51, 52], which limits the functionality and efficiency of enzymes, including those needed to repair and replicate DNA.

The presence of oxygen also increases the damage to lipids. Indeed, unsaturated lipids are liable to undergo a process known as lipid peroxidation, a chain reaction, initiated by reactive free radicals such as ^·^OH, which, in the presence of oxygen, leads to the formation of lipid peroxides and other derivatives [53]. Peroxidation of membrane lipids alters their structure and interferes with membrane function. Studies beginning in the 1960s showed that several halogenated hydrocarbons exert their toxic effects by stimulating lipid peroxidation and gave early emphasis on the biological role of free radical reactions [54–56]. However, although detection of end products of lipid peroxidation is the evidence more frequently quoted for a role of free radicals in human disease or tissue injury by toxins, lipid peroxidation has not been well exploited to understand the biological effects of radiation. Probably, this occurred because increases in protein and DNA damage are often more important events in causing cell injury than peroxidation of membrane lipids, which is often a late event accompanying rather than causing final cell death.

## 6. Oxygen Toxicity

For a large part of living beings, oxygen is an essential molecule for survival, being the basis of biological oxidations, which meet most of the energy needs of aerobic organisms. Although oxygen is essential for these organisms, it can also act as a toxic agent and pose a threat to their existence. This contradictory aspect of aerobic life was defined by Davies as the “paradox of aerobic life” [57]. All organisms can survive in the presence of oxygen because, in adapting to the oxidizing atmosphere of the earth, they have developed an elaborate antioxidant defence system. However, this system is only suitable for oxygen pressure in the atmosphere (about 156 mmHg), and it is widely demonstrated that exposure to oxygen pressures greater than the atmospheric one causes severe damage.

Historically, reports of harmful effects of oxygen followed soon after the discovery and purification of the gas in the late 18th century, which permitted scientists to expose animals to oxygen-enriched atmospheres.

Usually, priority in the discovery of oxygen is given to Priestley who called it “dephlogisticated air” [58], even though the oxygen had been discovered in 1772 by Carl W. Scheele, a Swedish apothecary, who, however, did not publish his findings until 1777 [59].

Anyway, Priestley was one of the first to suggest that there may be adverse effects of this “pure air” when he observed a candle burn out faster in oxygen than in air and wondered if “the animal powers are too soon exhausted in this pure kind of air” [58].

On the other hand, the toxicity of atmospheric oxygen had already been exploited by our ancestors for therapeutic purposes, such as the treatment, by exposure to air, of sites infected with anaerobic bacteria.

Immediately after the promulgation of the combustion theory of respiration, the pertinent literature recorded a considerable number of investigations in connection with the question of oxygen toxicity. According to various authors, definite effects were obtained by breathing pure oxygen. The respiratory exchange was increased, the circulation quickened, and congestion of the lungs, or even inflammation and death, occurred. The theory was that the addition of oxygen increased the pulmonary combustion and thereby produced these pathological changes. This result was controverted by Regnault and Reiset in their classical investigation [60]. They showed that no increase in oxidation occurred and no pathological changes ensued on the exposure of animals to atmospheres rich in oxygen.

The first important contribution in the field of oxygen toxicity was by Paul Bert who, in 1878, first demonstrated convulsions in larks exposed to 15-20 ATA (atmosphere absolute) air [61], and the toxic effects of oxygen on the central nervous system are hence called “Paul Bert effect.” Furthermore, in a large series of experiments, he showed that the effects on all living organisms arising from variations in barometric pressure are entirely the result of the tensions at which the oxygen is maintained in the various atmospheres. By exposing an animal to four atmospheres of oxygen, the same effect is brought about as that caused by increasing the barometric pressure of the air 20 times [61].

In 1899, J Lorain Smith, while trying to reproduce “Bert effect,” noticed fatal pneumonia in rats after 4 days of exposure to 73% oxygen at 1 ATA [62]. This marked the discovery of pulmonary toxicity of oxygen, called the “Lorrain Smith effect.”

The signs of oxygen toxicity are detectable in various tissues, even if the most worrying ones are those of the central nervous system and of the lung, which can be considered real target organs.

However, when after 1940 the spread of a syndrome currently called “retinopathy of prematurity” was found in preterm infants, this syndrome was not related to oxygen toxicity. Retinopathy was characterized by the formation of new abnormal blood vessels in the retinal periphery and could result in real blindness due to retinal detachment. Furthermore, the lower the gestation time and the weight of the newborn at birth, the greater the risk. Retinopathy initially described as retrolental fibroplasia by Terry in 1942 [63] was the leading cause of blindness in children in the United States. Only in 1951, it was suggested that the retinopathy was due to an excess of oxygen in the incubators where premature infants were placed to promote their development [64].

### 6.1. Mechanisms of Oxygen Toxicity

The first hypothesis made to explain the toxic effects of oxygen was that it inhibits cellular enzymes. There were several examples of enzymatic inactivation linked to oxygen in anaerobic organisms [65]. However, there was a reluctance to conclude that the inhibitory effects of oxygen on metabolism were the direct cause of the symptoms of the oxygen toxicity on the intact animal.

Haugaard, who reviewed the pertinent literature in 1968 [66], gave two reasons why the hypothesis did not gain wider acceptance. First, the rates at which enzymes were inactivated in vitro were too slow to account for the rapidity of toxic effects in intact animals. Furthermore, in aerobic cells, most of the enzymes are completely insensitive to oxygen, and those inactivated are quite low.

Despite being a free radical, O_2_ does not present a high reactivity. The reactions in which it is involved do not normally occur at ordinary temperatures or in the absence of catalysts, although its high oxidizing power makes most of the substances of biological interest thermodynamically unstable in its presence. This apparent contradiction is explained by the electronic configuration of the O_2_ in the ground state, which has two electrons with parallel spins in the two outermost orbitals. For this reason, in the oxidation processes, it would be necessary to make available, by the molecules to be oxidized, two electrons with spin parallel to each other and opposite those of the unpaired electrons of oxygen. Since the molecules of stable organic compounds contain valence electrons with opposite spin, the need to operate spin inversion before electrons are accepted in the oxygen orbitals slows or precludes the reaction with such molecules, a phenomenon called spin restriction [67].

Since the energy necessary for vital processes in aerobic organisms derives from oxidation reactions in which oxygen is consumed, it is evident that in such organisms, processes are working through which the spin restriction is, in some way, eliminated with consequent increase of the reactivity of oxygen.

It is possible to put enough energy into oxygen to elevate one of its parallel spinning electrons to a higher orbital and in the process to invert its spin. Such an excited state of oxygen is referred to as singlet oxygen, and for singlet oxygen, the spin restriction has been eliminated and it is much more reactive than is ground state oxygen [67].

The energy inherent in visible light is enough to convert ground state oxygen into singlet oxygen, but oxygen does not absorb visible light. Dyes such as methylene blue or rose Bengal do absorb visible light and then, upon collision with oxygen, can transfer the energy from that light to oxygen. That is one basis of photosensitized oxidations. There is a way that the spin restriction can be circumvented, and that is by adding the electrons to oxygen one at a time at a rate that allows electronic spin inversions to occur between collisional events. The univalent pathway of oxygen reduction requires that intermediates of oxygen reduction be generated, which are reactive and can damage biological molecules.

On this basis, an important turning point in understanding the mechanism of toxicity occurred when in 1954 Gerschman and associates published an article hypothesizing that oxygen poisoning and radiation injury have at least one common basis of action, possibly through the formation of oxidizing free radicals [68]. The hypothesis was based on similarities of the effects of irradiation and exposure to hyperbaric oxygen, on the synergism between radiation and hyperbaric O_2_ in decreasing the survival of exposed mice, and on observations that substances of varied chemical nature known to increase resistance to irradiation exhibited protective action against oxygen poisoning.

In the same year, Commoner et al. made the first observation of free radicals in biological systems using electron paramagnetic resonance methods [69].

Two years later, Harman hypothesized that oxygen radicals may be formed as by-products of enzymatic reactions in vivo. He proposed that traces of iron would catalyse oxidative reactions in vivo and that peroxidative chain reactions were possible by analogy to the principle of in vitro polymer chemistry. Harman also implicated that free radicals are produced during aerobic respiration in cellular damage, mutagenesis, cancer, and degenerative process of biological aging [70].

The theory then led to a significant corollary on the role of antioxidants: if the oxidative reactions are a chain, one molecule that intercepts the radical initiator or propagator prevents the entire chain. It follows that only the reactions of radicals that trigger chains may be the subject of a functionally valid inhibition by antioxidants: a concept that, although simple, is today very often forgotten.

## 7. The Superoxide Dismutase Discovery

Harman's idea did not capture the imagination of most life scientists, until the discovery of the superoxide dismutase (SOD) enzyme by McCord and Fridovich [71]. Toward the end of the 60s, these authors investigated the oxygen-dependent reduction of cytochrome c by xanthine oxidase [72]. Oxygen would be expected to oxidize cytochrome c rather than to facilitate reduction, so that McCord and Fridovich suggested that xanthine generated an unstable reduced form of oxygen, presumably the superoxide anion, and that this radical was the agent which directly reduced cytochrome c. Because some proteins inhibited the reduction of cytochrome c by xanthine oxidase, they realized that inhibitory effects were due to novel protein contaminating the other proteins tested, which catalysed the superoxide dismutation. They purified from bovine erythrocytes a protein that inhibited the reduction of cytochrome c. This protein had been previously purified as a copper-binding protein [73], but McCord and Fridovich showed that this protein was an efficient scavenger of superoxide, thus identifying the first enzyme known to act on a free radical substrate [71].

Similar enzymes were soon recovered from a wide range of air-tolerant bacteria but, significantly, were scarce in anaerobes [74].

A protein antioxidant catalyst which had as its substrate a small inorganic free radical was something novel to biochemistry at that time, but, paradoxically, the observation of the widespread distribution of SOD family of enzymes in cells was the major hint of the wide occurrence of free radical activity in the body.

Despite this, initially, the role of SOD was greeted with scepticism by some. Not all were prepared to accept that SOD was exclusively a biological enzyme and preferred to view it more as a copper transport protein.

In these conflicting opinions, an important role played the idea that the radicals were too reactive and uncontrollable to participate in any reaction involving an enzyme.

However, the discovery of the superoxide dismutase made real the existence of radicals in living systems and led to the “superoxide theory,” which foresaw that the O_2_ toxicity was due to the generation of anion radical superoxide [74]. Interestingly, subsequent observations performed modifying SOD activity showed that superoxide was at least partially responsible for the mechanisms leading to the well-known effects of oxygen on radiosensitization [75].

Subsequent studies showed that the superoxide is a moderately reactive radical, which under physiological conditions generally behaves as a mild reductant rather than an oxidizing agent. This is because superoxide exists naturally as a small anion, which is more prone to give its electron than to accept a second electron from another biological molecule. The limited chemical reactivity of superoxide created considerable controversy about the role of superoxide in cellular toxicity [76].

However, superoxide can be considered the “primary” oxidant species, whose generation can lead to the formation of more reactive “secondary oxidant species” [77].

To explain how a more potent oxidant might be generated by superoxide, the iron-catalysed formation of hydroxyl radical from hydrogen peroxide was proposed. Indeed, superoxide can undergo SOD-catalysed dismutation, producing hydrogen peroxide:

(7) $$\begin{array}{c} 2{{\text{O}}_{2}}^{\cdot -}\overset{\text{SOD}}{\to }{\text{O}}_{2}+{\text{H}}_{2}{\text{O}}_{2} \end{array}$$

Hydrogen peroxide reactivity is reduced by the stability of its oxygen-oxygen bond. However, in the presence of reduced transition metals (e.g., Fe^2+^ or Cu^+^), it can be converted into the hydroxyl radical (Fenton reaction) (Equation (4)). Conversely, the hydroxyl radical has high reactivity, which makes it a very dangerous radical with a very short in vivo half-life of approx. 10^−9^ s [78].

The hydroxyl radical oxidizes most organic molecules at diffusion-limited rates, but its most significant impact is likely to be upon DNA since even a single DNA lesion is potentially mutagenic or lethal.

Because of its strong oxidant capacity and importance in radiation-induced damage to biological molecules, hydroxyl radical became widely accepted as the major toxin produced in vivo.

In the last quarter of the 20th century, the scientific community began to accept the fundamental principle that many of the same free radicals that were formed as a result of ionizing radiation interacting with biological material were also formed as by-products of oxidative metabolism.

## 8. Reactive Oxygen Species

It is worth noting that until the mid-1970s, the literature almost exclusively refers to free radicals. Later, it became evident that not only free radicals but also nonradical products, such as H_2_O_2_ or hypochlorous acid (HOCl), which are also powerful oxidizing agents, participate in free radical reactions. To consider both the radical and the nonradical species, the more general term “reactive oxygen species” (ROS) was introduced [79]. Later, species containing nitrogen, such as nitric oxide (NO^·^) and peroxynitrite (ONOO^−^), were shown to be biological molecules and were termed reactive nitrogen species (RNS) [80].

This view that ROS were generated in biological systems was also supported by the finding of the cellular sources of such species. A mitochondrial H_2_O_2_ production had been first observed in 1966 by Jensen, who found that antimycin-insensitive oxidation of nicotinamide adenine dinucleotide (NADH) and succinate by bovine heart submitochondrial particle was coupled with H_2_O_2_ production [81]. Soon after, it was demonstrated, for the first time, that under aerobic condition, H_2_O_2_ was generated by pigeon heart mitochondria in the presence of succinate [82]. The discovery that mitochondria contain their own SOD, MnSOD [83], and the subsequent detection of the mitochondrial generation of superoxide radical anion [84] confirmed that H_2_O_2_ generated within mitochondria arose from the dismutation of superoxide (O_2_^·−^) and the biological significance of mitochondrial O_2_^·−^ production. Since then, a huge literature has developed on the sources and consequences of mitochondrial production of reactive oxygen species. In particular, the discovery that electron transfer along the inner mitochondrial membrane carriers is associated with the formation of reactive oxygen species suggested the mitochondrial involvement in degenerative processes linked to several diseases and aging.

In this regard, it is worth noting that a detailed study evaluating the variations of formation of H_2_O_2_ under different metabolic conditions showed a marked increase in such a formation and its immediate onset under hyperbaric conditions [85], thus providing an explanation at the molecular level for “O_2_ poisoning.”

Although mitochondria are responsible for the continuous production of reactive oxygen species, they are not the only source at either the organism or the cellular level. In fact, in the 70s, evidence was already available that generation of active oxygen species can also occur as a by-product of other biological reactions in other cellular organelles.

For example, it was long known that liver microsomes contained an enzyme system, NADPH (TPNH) oxidase, which catalyses the oxidation of NADPH by oxygen to yield NAD^+^ and hydrogen peroxide [86]. In later years, this result was confirmed repeatedly, and the H_2_O_2_ formation was believed to be linked to the monooxygenation system for xenobiotics since pretreatment of the animals with phenobarbital increased the rate of H_2_O_2_ formation [87] and inhibitors of cytochrome P450 affected the production of H_2_O_2_ [88].

It was also recognized that NADPH-dependent redox chain of microsomes generated hydrogen peroxide and superoxide radicals [89], the latter being possibly precursors of the former. Moreover, superoxide formation involved autooxidation of NADP-specific flavoprotein dehydrogenase (NADPH-cytochrome c reductase) [88] and dissociation of oxycomplex of cytochrome P450 [90].

Other examples of cellular organelles which were found to have high oxidative activity include peroxisomes, which contain oxidases, which reduce oxygen to hydrogen peroxide at the expense of the oxidation of a substrate RH_2_, and large amounts of catalase (CAT), an enzyme able to reduce hydrogen peroxide to water [91]. The idea that the peroxisomes generate ROS as an integral feature of their normal metabolism was further exemplified by the fact that peroxisomes in the rat liver may be responsible for as much as 20% of the oxygen consumption and 35% of the H_2_O_2_ production [92].

In 1961, Iyer et al. [93] had shown that the phagocyte respiratory burst results in the generation of hydrogen peroxide; in 1964, Rossi and Zatti [94] had correctly proposed that an NADPH oxidase was responsible for the respiratory burst; and in 1973, Babior et al. [95] reported that the initial product of the respiratory burst oxidase was superoxide and not hydrogen peroxide. The phagocyte NADPH oxidase was the first identified example of a system that generates ROS not as a by-product but rather as the primary function of the enzyme system.

The next steps were the identification of proteins responsible for ROS production in phagocytes [96] and the cloning of the gene coding for the catalytic subunit of the phagocyte NADPH oxidase, commonly referred to as gp91^phox^ [97], which in the novel NOX terminology was called NOX2. In parallel with the progress toward understanding the phagocyte NADPH oxidase, a series of observations suggested that enzyme systems like the phagocyte NADPH oxidase exist in many other cell types (see Bedard and Krause [98] for a review).

Several soluble cell components, including thiols, hydroquinones, catecholamines, and flavins, were found to be able to undergo redox reactions and contribute to intracellular ROS production [99]. To these, it occurs to add several cytosolic enzymes that produce ROS during their catalytic activity. Among the enzymes producing ROS, the researchers paid close attention to xanthine oxidase (XOR).

XOR is a flavoenzyme, which was extensively studied for its biochemical and structural properties ever since 1902 [100]. The enzyme catalyses the oxidation of hypoxanthine to xanthine and xanthine to uric acid using NADP^+^ or O_2_ as an electron acceptor. The mammalian enzymes exist in the NAD^+^-dependent form (xanthine dehydrogenase (XDH)) in freshly prepared samples from organs under normal conditions, i.e., they exhibit high xanthine/NAD^+^ reductase activity, even in the presence of O_2_ [101]. In oxidatively damaged tissues, the XDH is converted, due to proteolysis or oxidation of the thiol groups of cysteine, into xanthine oxidase (XO) [101], which, when acting on its substrates, is able to transfer electrons to molecular oxygen generating the superoxide [72].

Some years after, it was proposed that xanthine oxidase-derived oxidants mediate the microvascular injury associated with reperfusion of the ischemic intestine [102], and subsequently, this idea was extended to several organs and systems [103].

## 9. Reactive Nitrogen Species

The discussions above have focused on oxygen and oxygen-derived species. However, there are other relevant radicals, nitric oxide, NO^·^, and its derivatives.

Nitric oxide is the first gaseous species unequivocally identified as an endogenously generated cell signalling/effector agent. Furthermore, superoxide is considered the primary ROS from which more strongly oxidizing species originate, so nitric oxide is considered the primary RNS.

Nitric oxide, first identified as a gas by Joseph Priestley, is a simple molecule consisting of just one atom of oxygen and one atom of nitrogen [104]. For a long time after this discovery, NO^·^ was thought to be simply an atmospheric pollutant. It is produced above all during high-temperature combustion processes, like those that happen in the engines of cars, together with nitrogen dioxide (NO_2_^·^), another free radical. It is then oxidized in the atmosphere by oxygen and more rapidly by ozone-producing nitrogen dioxide. The toxicity of nitrogen dioxide is significant, contrary to that of nitric oxide, which is instead limited. Indeed, nitrogen dioxide plays a fundamental role in the formation of photochemical smog as it is the basic intermediate to produce a whole series of very dangerous secondary pollutants such as ozone, nitric acid, nitrous acid, alkyl nitrate, and peroxyacetyl nitrates.

The proposal that NO^·^ was a biological molecule was rather controversial because early indications of its presence in biological systems were ignored for many decades and even because nitrovasodilators had been used clinically for a century without understanding their mechanism of action. In fact, at that time, drugs such as nitroglycerine were given to patients for heart conditions like angina to promote vasodilation and reduce blood pressure, but no one knew how these drugs worked.

The discovery of NO^·^ as a biological molecule involved in vasodilation is linked to the studies dealing with cyclic guanosine monophosphate (cGMP) and endothelial-derived relaxing factor (EDRF).

Cyclic GMP began to emerge as a second messenger during the late 1960s and early 1970s. The existence of endogenously produced cGMP was demonstrated by its isolation and identification from rabbit urine [105]. Later, this was confirmed by another study from the same laboratory [106], which suggested that cGMP is synthesized in a reaction catalysed by a cyclase. In 1966, a phosphodiesterase specific for cGMP was isolated and partially purified from a dog heart [107], and subsequently, guanylyl cyclase activity was described [108]. Thus, it became apparent that steady-state levels of cGMP in cells and tissues were determined by the balance of cGMP synthesis by guanylyl cyclase and cGMP degradation by phosphodiesterases. Increased levels of cGMP produced by guanylate cyclase within vascular smooth muscle allowed blood vessels to relax and thus increase blood flow.

In 1977, two groups demonstrated independently that organic nitrates induced a dose-dependent increase in the levels of cGMP in smooth muscle [109, 110]. Subsequently, biochemical experiments showed that all the nitrovasodilators and nitric oxide (NO) activate the soluble guanylate cyclase [111, 112]. However, the possibility that NO might be synthesized in mammals and was able to function as an endogenous regulator was considered to be too far-fetched for another decade.

Progress was made when Furchgott and Zawadzki [113] found that without the endothelial cells the smooth muscle cells were not able to cause vasodilation. This suggested that a factor produced by the endothelial cells was required for relaxation of the blood vessels acting on an unidentified target in skeletal muscle. The diffusible factor later referred to as the endothelium-derived relaxing factor (EDRF) [114] was quickly inactivated by oxyhaemoglobin and was inherently unstable in the perfusion cascades used to study the vasorelaxation. It was soon shown that this mysterious factor, termed endothelial-derived relaxing factor (EDRF), increased cGMP synthesis in isolated blood vessels and increased protein phosphorylation in smooth muscle [115, 116].

Widespread speculation about the chemical nature of EDRF developed soon after its discovery [117, 118], but only in 1988, Furchgott [119] and Ignarro et al. [120] independently in papers presented at a symposium in Rochester, suggesting that EDRF produced by endothelium may be NO^·^.

Soon after the 1986 symposium, three laboratories compared the biological and chemical characteristics of EDRF and NO, and all found EDRF released upstream and NO infused upstream to have similar rates of decay, similar susceptibility to inhibitors like Hb and superoxide generators, and similar stabilization by SOD [121–123]. A year later, Palmer and coworkers [124] made the major finding that the source of endothelial NO was guanidinium hydrogen of L-arginine.

In 1990, Bredt and Snyder purified oxide nitric synthase (NOS), the enzyme responsible for NO synthesis from a rat cerebellum (NOS) [125]. Because this NOS was from the rat cerebellum, it was named the neuronal NOS isoform (nNOS or NOS-1). The discovery of constitutively expressed neuronal NOS was quickly followed by the identification of endothelium-derived NOS (eNOS or NOS-3), constitutively expressed [126], and inducible NOS (iNOS or NOS-2) not constitutively expressed [127].

The discovery of NO^·^ as an enzymatically generated free radical was paralleled by the recognition that it could readily react with O_2_^·-^ [128]. It was known that the combination reaction leads to proximities [129], a peroxy acid originally studied in the chemical literature as a strong oxidizing and nitrating compound [130]. A paper on the unusual properties of a mixture of hydrogen peroxide and nitrous acid by Baeyer and Villiger from 1901 [131] can be regarded as the first report on peroxynitrite. However, as an oxidant, peroxynitrite attracted little attention because it produced a bewildering array of products even with simple starting substrates such as phenol. In 1970, several investigators more thoroughly characterized the chemistry of peroxynitrite showing that it decomposed to form hydroxyl radical and nitrogen dioxide [132].

At the end of the 80s, when NO^·^ was discovered, the controversy about the reactivity of superoxide and its biological effects was still open. Therefore, it is not surprising that NO^·^ was viewed by some researchers as a protective factor because of its capacity to scavenge radical superoxide thus acting as an antioxidant [133, 134]. However, in 1990, Beckman et al. suggested that the reaction product of superoxide and nitric oxide was peroxynitrite, which decomposes when protonated to form the potent oxidants, hydroxyl radical and nitrogen dioxide [135]:

(8) $$\begin{array}{c} {{\text{O}}_{2}}^{\cdot -}+{\text{NO}}^{\cdot }⟶{\text{ONOO}}^{-}\\ {\text{ONOO}}^{-}+{\text{H}}^{+}⟶\text{ONOOH}⟶{\text{NO}}_{2}^{\cdot }{+}^{\cdot }\text{OH} \end{array}$$

These results were confirmed by Darley-Usmar and collaborators [136] using systems to cogenerate superoxide and NO^·^.

The reaction of NO^·^ with O_2_^·-^ occurs biologically even in the presence of superoxide dismutase (SOD), indicating that it is extremely fast to outcompete the enzyme-catalysed dismutation. Thus, nitric oxide may substantially increase the toxicity of superoxide converting a relatively mild reductant into at least two potent oxidants. The recognition that the homolysis of ONOOH could yield ^·^OH led to the postulation of a new biologically relevant mechanism of oxygen radical-mediated molecular damage, which is more effective than the widely accepted reaction of reduced iron with hydrogen peroxide (known as the Fenton reaction or the iron-catalysed Haber-Weiss reaction).

On the other hand, although Fenton chemistry was known to occur *in vitro*, its significance under physiological conditions was and remains a matter of debate today, noting particularly the negligible availability of “free catalytic iron” due to its effective sequestration by the various metal-binding proteins. However, organisms overloaded with iron (as in the conditions of haemochromatosis, *β*-thalassemia, and haemodialysis) contain higher amounts of “free available iron”, and this can have deleterious effects.

## 10. Oxidative and Nitrosative Damage

In the last quarter of the 20th century, following the discovery of the superoxide dismutase enzymes by McCord and Fridovich [71], the scientific community began to accept the fundamental principle that free radicals were formed as by-products of oxidative metabolism When ROS were initially integrated into biomedical concepts, it was thought that they caused exclusively toxic effects and were associated with pathologies. In fact, ROS avidly interact with many molecules including other small inorganic molecules as well as proteins, lipids, carbohydrates, and nucleic acids. Through such interactions, ROS may irreversibly destroy or alter the function of the target molecule.

Moreover, after its early description as an endothelial-derived relaxing factor, NO^·^ emerged not only as a fundamental signalling device but also as a potent mediator of cellular damage in a wide range of conditions, and nitrosative stress has been implicated in the pathogenesis of a large variety of disorders.

Although NO^·^ has often been described as highly toxic and reactive, it is not, and most of the cytotoxicity attributed to NO^·^ is rather due to peroxynitrite produced from the diffusion-controlled reaction between NO^·^ and superoxide anion. Indeed, although not a free radical in nature, peroxynitrite is much more reactive than its parent molecules NO^·^ and O_2_^·-^.

Kinetic studies indicated that peroxynitrite oxidized target molecules through two distinct mechanisms. First, peroxynitrite and its protonated form peroxynitrous acid (ONOOH) exerted direct oxidative modifications through one- or two-electron oxidation processes. Only a few chemical groups directly reacted with peroxynitrite, which favoured selective reactions with key moieties in proteins, such as thiols [137] and iron/sulphur centres [138]. The second mechanism involved peroxynitrite indirectly mediating oxidation by decomposing into highly reactive radicals. Indeed, peroxynitrite decomposition produces the highly reactive ^·^OH radicals, as well as nitrogen dioxide, which is a strong oxidant with significant cytotoxic potential [139, 140]. Nitrogen dioxide is also formed when peroxynitrite reacts with carbon dioxide. Such a reaction also leads to the formation of the radical anion carbonate (CO_3_^·-^):

(9) $$\begin{array}{c} {\text{ONOO}}^{-}+{\text{CO}}_{2}⟶{\text{CO}}_{3}^{\cdot -}+{\text{NO}}_{2}^{\cdot } \end{array}$$

Carbonate radical is more selective than hydroxyl radical but can initiate many of the damaging reactions commonly attributed to hydroxyl radical in the biological literature and is perhaps equally significant as a biological oxidant [141]. An important aspect of peroxynitrite-mediated toxicity is its capability of promoting protein tyrosine nitration through nitrogen dioxide and anion carbonate considered as a central aspect of peroxynitrite-mediated cytotoxicity [142].

### 10.1. Antioxidant System

Interestingly, the observations regarding free radicals were accompanied by the discovery that biological systems were equipped with an integrated antioxidant defence system capable of counteracting the damaging effects of ROS and RNS.

Catalase had been first noticed in 1818 by Louis Jacques Thénard, who discovered H_2_O_2_ and suggested that its breakdown was caused by an unknown substance [143], to which, in 1900, Oscar Loew gave the name catalase and found it in many plants and animals [144].

In 1957, Mills discovered an enzyme (GPX) using the reducing properties of glutathione for the protection of human erythrocytes against degradation of haemoglobin by hydrogen peroxide [145].

Previously, a heat-labile system capable of reducing GSSG was discovered in the liver by Hopkins and Elliott [146], and the next year, Mann [147] found that the hepatic GSSG reduction was linked to glucose oxidation by what was later identified as NADPH production in the pentose phosphate pathway. The enzyme directly involved in the reduction of GSSG, glutathione reductase (GR), was subsequently demonstrated in the rat liver by Rall and Lehninger [148]. Thus, it was realized that GPX and GR are the most important enzymes in maintaining cell redox homeostasis, since their combined action is the major determinant of reduced glutathione (GSH) content of tissues because GR serves to regenerate the active glutathione from its oxidized form produced in the GPX reaction.

Thioredoxin (Trx) was discovered in 1964 and characterized as a hydrogen donor for the enzymatic reduction of ribonucleotides in *Escherichia coli* [149]. The reduction reaction of thioredoxin required NADPH and was catalysed by a specific enzyme, which, in an accompanying work, was called thioredoxin reductase (TrxR) [150].

Subsequent investigation showed that the Trx/TrxR system is the major ubiquitous disulphide reductase responsible for maintaining proteins in their reduced state [151].

Until quite recently, CAT and GPX were thought to be the major peroxide-reducing enzymes protecting cells. The shift began in 1994 when protein sequence comparisons led to the recognition of a third abundant and widespread group of peroxidases [152]. The name peroxidoxins initially proposed for this group was quickly morphed to become the currently used peroxiredoxins (Prxs) [153]. These enzymes were quite distinct from catalases and GPXs especially in that they had no special cofactor but simply used cysteine residues for catalysis. Moreover, while for many Prxs the physiological reductant appears to be thioredoxin [152, 153], for some there exists a specific peroxiredoxin reductase (PrxR) that contains a thioredoxin-like domain [154]. As Prxs can also show high catalytic reactivity with peroxynitrite, they may also be important for defence against reactive nitrogen species [155].

## 11. Oxidative Stress

It is worth noting that in healthy organisms, the production of free radicals is low, and the antioxidant defence systems quickly remove ROS and the RNS before they cause structural and functional damage to the cell. The balance is not perfect so that some ROS and RNS-mediated damage occurs continuously, and damaged molecules must be repaired or replaced. This is demonstrated by the finding of ongoing oxidative damage in vivo in animals, including humans. For example, low levels of oxidative base damage products are present in DNA isolated from all aerobic cells. Low levels of carbonyls and certain products resulting from the attack of ROS upon proteins are detected in healthy animal tissues and body fluids. Age pigments accumulate in tissues, and specific end products of lipid peroxidation are present in body fluids [156]. These observations indicated that ROS-removing systems keep cellular levels of ROS in tissues rather than removing them completely. Moreover, it was understood that, in addition to exposure to high oxygen pressure and ionizing radiation, there were several different pathophysiological conditions in which the imbalance between the speed of production of free radicals and the capacity of the cellular defence systems becomes wider.

In 1985, Sies introduced the term “oxidative stress” to indicate this imbalance between prooxidants and antioxidants in favour of the former [157]. Severe oxidative stress produces DNA damage, rises in intracellular free Ca^2+^ and iron, damage to proteins (including membrane ion transporters), and lipid peroxidation.

Among the conditions that lead to the establishment of oxidative stress, there are, to name just a few, carbon tetrachloride toxicity; ischemia-reperfusion injury; hypermetabolic states, such as hyperthyroidism; and physical activity.

### 11.1. Carbon Tetrachloride Toxicity

It was long known that inhalation of vapours of carbon tetrachloride (CCl_4_), a chlorinated hydrocarbon used as a solvent for oils and fats, as a refrigerant and as a dry-cleaning agent could depress central nervous system activity and cause degeneration of the liver and kidneys [158]. However, there was a paucity of information concerning mechanisms of its action. Only in 1961, it was proposed that the hepatotoxic effects of CCl_4_ were due to the free radicals formed by homolytic scission of the carbon halogen bond [159]. In 1975, the existence of free radicals during CC1_4_ metabolism was proven by an electron spin resonance study [160]. Subsequent investigation showed that reductive dehalogenation of CCl_4_ was catalysed by cytochrome P450, the terminal oxidase of the hepatic mixed-function oxidase system [161]. Moreover, it was found that low partial pressure of oxygen in tissue resulted in the predominant formation of CC1_3_^·^ and CHC1_2_^·^ radicals, whereas high partial pressure of oxygen shifts CC1_4_ metabolism toward the formation of the CC1_3_OO^·^ radical with consequent lipid peroxidation [162].

### 11.2. Ischemia-Reperfusion Injury

It is long known that, although restoration of blood flow is the sole method for salvaging ischemic tissues, the extent of injury often increases when the blood supply is restored. One example of reperfusion injury is the phenomenon of stunning that was described by Heyndrickx and colleagues in the dog heart in 1975 [163]. They showed that a myocardium reversibly injured by ischemia does not contract as efficiently as the control myocardium after reperfusion. The finding of the paradoxical enhancement of the injury response following reperfusion (or reoxygenation) of ischemic (or hypoxic) tissue led to the proposal that the sudden reintroduction of molecular oxygen to energy (and oxygen)-starved tissue results in a unique type of injury response that is not manifested during the period of hypoxic stress [164, 165]. Since its inception, the concept of reperfusion injury steadily gained attention likely for the implication of this mechanism of tissue injury in a growing list of organs. In the early 1980s, ROS were proposed as potential mediators of reperfusion injury because of the detection of chemical products generated by the reaction of ROS [166]. The idea that ROS could account for reperfusion injury was quickly embraced, mainly because it was consistent with the observation that interventions that enhanced ROS scavenging and/or detoxification protected against reperfusion injury following intestinal [102], myocardial [167, 168], and skeletal muscle [169] ischemia.

The oxidative stress elicited in tissues/cells following ischemia-reperfusion was linked to a variety of different sources of ROS, including xanthine oxidase, NADPH oxidase, and mitochondria. The hypothesis that xanthine oxidase is a major source of ROS was initially proposed by Shlafer et al. [167], which predicted that the accumulated hypoxanthine, arising from ATP metabolism, would react with the readmitted oxygen to produce a burst of superoxide, because of the conversion of the xanthine dehydrogenase to xanthine oxidase [170].

NADPH oxidase is another source that accounts for an important part of the ROS formed during ischemia-reperfusion.

Studies implicating neutrophils in reperfusion injury provided some of the earliest evidence suggesting the involvement of NOX as a source of ROS in postischemic tissue [171, 172]. However, there are several lines of evidence that support a role for nonphagocytic NOX as a source of ROS following I/R [173].

Mitochondria have been implicated as a major source of I/R-induced ROS production in a variety of organs. The proposal that the respiratory chain is a major source of ROS during reperfusion of the ischemic myocardium [174] was supported by the observation that a generation of oxygen radicals was induced *in vitro* upon reoxygenation of mitochondria isolated from hearts that had been subjected to ischemia [175]. Further support was obtained demonstrating by electron paramagnetic resonance that resumption of mitochondrial oxidative phosphorylation upon postischemic reflow can be a source of oxygen radicals in intact rabbit hearts [176]. It was proposed that, upon the respiration resumption, ROS generation is promoted by O_2_ interaction with ubisemiquinone, which accumulates in mitochondria during ischemia because of respiratory chain inhibition [177]. The observation that the increase in ischemia duration was directly related to a gradual increase in lipid peroxidation and decline in mitochondrial respiration and inversely related to functional recovery of the tissue [178] supported the idea that heart performance is strongly conditioned by mitochondrial functionality. Further support was provided by the observation that the antioxidant protection of mitochondrial function was associated with decreased impairment of cardiac function following ischemia-reperfusion [179].

### 11.3. Hyperthyroidism

It is long known that thyroid hormones are key regulators of growth, development, and metabolism. It is also well known that elevated circulating levels of thyroid hormones are associated with modifications in the whole organism and several body districts. The most studied modification found in hyperthyroid animals is the increase in their basal metabolic rate (BMR) due to an increase in the rate of O_2_ consumption in target tissues. The idea is well established that, like other long-term effects, thyroid calorigenesis is achieved by thyroid hormone influence on transcription of T_3_-responsive genes, which are mediated through nuclear thyroid hormone receptors (TRs) [180].

In the 80s, it was found that the hypermetabolic state in hyperthyroidism is associated with increases in lipid peroxidation in the rat liver [181], heart, and skeletal muscle [182]. Subsequent work demonstrated that products of lipid, protein, and DNA oxidation were found in several target tissues (see Di Meo and Venditti [183]for a review). More recent works showed stimulation of mitochondrial H_2_O_2_ generation following T_3_ treatment in the rat liver, heart, and skeletal muscle [184–186].

It is worth noting that the long-term effects of thyroid hormone on mitochondrial respiration are obtained by increasing the content of electron carriers, such as cytochromes [187, 188] and ubiquinone [189, 190], and their percent reduction [188, 191]. There is also indirect evidence that even mitochondrial concentration of oxidizable electron carriers increases in hyperthyroid tissues [184, 185]. Thus, it is conceivable that the increase in mitochondrial ROS generation, underlying cellular oxidative damage, is a side effect of the thyroid hormone-induced biochemical changes by which animal tissues increase their metabolic capacity.

Moreover, significant evidence that tissue oxidative stress underlies some dysfunctions produced by hyperthyroidism has been obtained exploring the application of antioxidants, in particular, vitamin E, as therapeutic agents in thyroid-related disorders (see Venditti et al. [192]for a review).

### 11.4. Physical Activity

As regards the physical activity, we all had the experience that any form of exercise, if carried out vigorously enough, can become painful. But only one form of exercise, eccentric exercise, if unaccustomed to it, leaves us stiff and sore the next day. This is because acute exercise could produce significant damage, including structural and functional alterations in skeletal muscle as well as in other tissues. According to some reports, mitochondrial swelling is provoked in rat gastrocnemius muscle by running [193], and extensive mitochondrial swelling is provoked in the myocardium of both running and swimming rats [194]. Thus, an increase in the total area occupied by mitochondria and sarcoplasmic reticulum is found in middle gluteal muscle from treadmill-exercised horses [195].

Moreover, the extent of tissue damage mainly depends on the intensity and duration of exercise [184]. Serum elevation of creatine kinase and lactate dehydrogenase, universally accepted as a marker of tissue damage, is found in runners following an 80 km race [196].

Skeletal muscle injury also depends on the way how the muscle is used, because the contraction-induced injury is much more severe following eccentric rather than concentric or isometric contraction [197].

Although several metabolic factors have been proposed as mechanisms of primary damage during acute exercise, in the late 1970s, it was proposed that ROS can be implicated in the damage that develops in the tissues owing to long-lasting aerobic exercise [198], even though, until now, direct evidence of ROS production during physical activity increase is lacking. The first demonstration that an increase of free radicals verified in both muscle and liver of rat subjected to an exhaustive running was obtained by Davies et al. [199] using electron spin resonance. After which, with the same method, it was reported that exhaustive endurance exercise also increases the signal related to free radical generation in the rat heart [200] and in human serum [201]. Final confirmation that free radical production is enhanced by high muscular activity was obtained in experiments performed stimulating electrically skeletal muscles [202].

Muscle contraction also affects NO production as first demonstrated by the increase in NO release from muscles exposed to prior electrical stimulation [203] and NO^·^ concentrations in expired air during physical activity [204].

The changes in the content of the markers of lipid, protein, and DNA and in the redox state after acute exercise supply indirect information on the enhanced ROS production during acute exercise.

Ever since Dillard and his colleagues showed that in a subject exercised on a bicycle ergometer there is an increase in the exhaled air content of pentane, an index of lipid peroxidation [198], the information on the formation of oxidative damage markers after exercise in the tissues of various animals, among which humans, has increased [205, 206].

An important practical consequence of the demonstration that free radicals are involved in tissue damage caused by exhaustive exercise is that it is possible to minimize the effect of such radicals by the administration of antioxidants such as beta carotenes, vitamin C, vitamin E, glutathione, or N-acetylcysteine.

Since Brady et al. reported in 1979 that a 4-week vitamin E-reinforced diet (50 IU/kg diet) inhibited increases in rat liver TBARS levels immediately after exhaustive swimming exercise [207], the inhibitory effect of antioxidant intake on exercise-induced oxidative damage has been studied extensively. Most research has demonstrated that antioxidant administration has beneficial effects against the damaging effects of intense physical exercise (see Kawamura and Muraoka [208] for a review).

Apart from the protective role against damage caused by free radicals during exhaustive exercise, antioxidants might have a positive effect on performance and the prevention of fatigue. In 1994, Reid et al. used NAC, a precursor of GSH, to investigate the effects of free radicals on muscle fatigue and found that intravenous infusion of NAC inhibited tibialis anterior muscle fatigue induced by low-frequency electrical stimulation [209]. This study was the first human study to prove that free radicals can induce muscle fatigue and that supplementary intake of antioxidants can reverse it.

Based on these studies, nutritional interventions and antioxidant integration began to be used frequently in athletic populations to reduce oxidative damage, ameliorate the performance, and accelerate the recovery of muscle function.

## 12. Free Radicals and Diseases

Owing to increased understanding of the damaging effects of ROS, the concept of ROS as agents of cellular damage in biological organisms became widely accepted in theories of aging. Moreover, a growing number of diseases/disorders were gradually linked either directly or indirectly with ROS. That is, while some of these disorders are primarily due to free radicals, others may be only secondarily involved. In this latter group, tissue injured by various processes, such as trauma, toxic substances, and infectious processes, may undergo free radical damage more rapidly than healthy tissues. Tissue destruction and degeneration can result in increased oxidant damage, by such processes as metal ion release, phagocyte activation, and disruption of mitochondrial electron transport chains (so that more electrons “escape” to oxygen to form O_2_^·-^). It follows that almost any disease is likely to be accompanied by increased formation of reactive oxygen species.

The protective effects of iron chelation in several disorders [210], superoxide dismutase and catalase in ischemia-reperfusion [170], and the protection by vitamins, such as vitamin C [211, 212], and antioxidants, such as probucol [213], under a variety of experimental conditions clearly indicated a key role for free radicals in many disorders. The list of diseases in which the ROS formation was implicated was already long in 1987 (it contained over 50 diseases) [214], and it was destined to grow in the following years.

In Table 3, several human diseases/disorders are reported for which a role for oxidative stress has been suggested. Among these disorders, the aging process has been included. More than 300 theories have been proposed to explain the aging process [215], but none has yet been generally accepted by gerontologists. However, the initial proposal by Harman that free radicals are causally related to the basic aging process [70] has received increasing acceptance as a possible explanation of the chemical reactions at the basis of aging [216].

The identification of free radical reactions as promoters of the aging process implies that interventions aimed at limiting or inhibiting them should be able to reduce the rate of formation of aging changes with a consequent reduction of the aging rate and disease pathogenesis. However, even though dietary antioxidants appear to be important in delaying/preventing certain human diseases, especially cardiovascular disease and some types of cancer [217], available evidence does not allow to recommend antioxidant supplementation as a useful means to prevent age-related pathophysiological modifications and clinical conditions. Furthermore, several concerns are present not only about their efficacy but also on their safety.

## 13. Useful Effects of ROS

Although free radicals were considered harmful to the living organism for their damaging effects, it was long known that these effects could be exploited for the benefit of the organism.

## 14. Radiation Therapy

As previously mentioned, during early practical work and scientific investigation, experimenters noticed that prolonged exposure to X-rays created inflammation and, more rarely, tissue damage on the skin. The biological effect attracted the interest of several physicians, among which Emil Grubbe, who, only a month or two after Röentgen's announcement also before understanding the physical properties and biological effects of X-rays, was the first physician known to use them for cancer treatment [218]. Interestingly, at that time, Grubbe was a student at the Hahnemann Medical College of Chicago. Because radiation was determined to be the cause of tumours, one of the professors at the homeopathic college suggested its therapeutic use in cancers. Thus, this incident has been considered by the proponents of homeopathic medicine as a further example from history in which an insight from a homeopathic perspective has provided an important breakthrough in medical treatment [219].

Radiation therapy uses particles or waves moving at a high frequency to target the DNA of cancer cells in the body and change the way they can replicate. Malignant tumours are characterized by unlimited cellular proliferation intimately connected with nucleic acid synthesis. If the DNA required for mitosis and replication is damaged, the cells are unable to replicate as usual and the growth of a cancerous tumour is inhibited. Of course, the mechanisms of the harmful effects of therapeutic exposure to ionizing radiation on tumour cells are the same as the effects of nontherapeutic exposure to radiation on normal cells. Therefore, radiation-induced ionizations can act directly on the cellular molecules and cause damage and can act indirectly producing free radicals which are derived from the ionization or excitation of the water component. Thus, hydroxyl radical generation is, paradoxically, the major mechanism by which malignant cells are killed during radiotherapy.

## 15. Phagocytosis

Another demonstration that free radicals could perform useful functions derived from the discovery that phagocytes (neutrophils, macrophages, and monocytes) release free radicals to destroy invading pathogenic microbes as part of the body's defence mechanism against disease.

In normal phagocytosis, polymorphonuclear leukocytes and macrophages consume large quantities of oxygen when engulfing their prey. This “extra respiration of phagocytosis” had already been described by the first half of the 20th century [220]. However, the unusual nature of the process was only revealed in 1959 when it was discovered that it was not inhibited by classical mitochondrial poisons such as cyanide and azide [221] indicating that it was not simply a reflection of the enhanced energy requirements of phagocytosis. In 1964, Rossi and Zatti [94] correctly proposed that an NADPH oxidase was responsible for the respiratory burst, which was soon discovered to be a requirement for the efficient killing of bacteria by neutrophils [222]. This observation was rapidly reinforced by the recognition that a syndrome identified by Berendes et al. in 1957 [223] “fatal granulomatosis of childhood” (now referred to as chronic granulomatous disease) (CGD), which was characterized by a severe predisposition to pyogenic infection, was associated with the complete absence of this oxidase activity from the patient phagocytes [224].

In 1973, soon after its discovery, superoxide dismutase was used to show that the product generated by activated neutrophils was superoxide [95]:

(10) $$\begin{array}{c} \text{NADPH}+{\text{O}}_{2}\overset{\text{NOX}2}{\to }\text{NADP}+{\text{O}}_{2}^{-}+{\text{H}}^{+} \end{array}$$

Moreover, the next year, it was demonstrated that this process was lacking in CGD [225].

Thus, the importance of ROS production by the immune system was clearly exemplified by multiple and persistent infections arising in patients with CGD in which the defective membrane-bound NADPH oxidase system made them unable to produce the superoxide anion radical (O_2_^·-^) and therefore to kill bacteria.

This important development provided a direct link between free radical chemistry and biology. At the time, most free radical chemistry was conducted by radiation biologists in test tubes, and its application to biology was purely theoretical. This new discovery was thought to prove that the production of free radical reactions in a biological process was toxic enough to kill organic structures as tough as bacterial and fungal spores. Soon, these observations were extrapolated to implicate free radical reactions in a host of pathological processes involving neutrophil infiltration and tissue damage

Subsequently, several additional highly reactive oxygen-derived metabolites, including hydroxyl radical, singlet oxygen (^1^O_2_), and hypochlorous acid (HClO), were identified or predicted to exist as a result of activation of phagocytic cells [226].

The observation that SOD, CAT, and ^·^OH scavengers, such as benzoate and mannitol, inhibited the phagocytic killing suggested that both O_2_^·-^ and H_2_O_2_ were required for the phagocytic bactericidal event and that ^·^OH generated by the interaction of O_2_^·-^ and H_2_O_2_ in the Haber-Weiss reaction was the toxic agent for microorganisms [227].

On the other hand, another possible explanation for the requirement for both O_2_^·-^ and H_2_O_2_ in the phagocytic activity might involve ^1^O_2_, which could be formed by a reaction between O_2_^·-^ and ^·^OH:

(11) O2·−+O·H⟶OH−+O12

The role of ^1^O_2_ in the killing of phagocytized bacteria had been suggested by the finding that *Sarcina lutea* containing carotenoid, a scavenger of ^1^O_2_, were protected against phagocytic bactericidal activity compared to a colourless mutant [228].

However, other reactive metabolites can be formed as a result of the metabolism of H_2_O_2_ by cellular enzymatic systems. The cytotoxicity of hydrogen peroxide itself is considerably enhanced in the presence of myeloperoxidase (MPO), which is simultaneously released from the azurophil granules into the phagocytic vacuoles. The enzyme-hydrogen peroxide complex that is formed can oxidize various halides to produce, for example, HClO, which has a potent bactericidal action [229]:

(12) $$\begin{array}{c} {\text{H}}_{2}{\text{O}}_{2}+{\text{Cl}}^{-}+{\text{H}}^{+}\overset{\text{MPO}}{\to }{\text{H}}_{2}\text{O}+\text{HClO} \end{array}$$

Singlet oxygen can, in turn, be formed by the interaction of H_2_O_2_ with hypochlorite [230]:

(13) HOCl+H2O2⟶H2O+HCl+O12

About a decade later, it was discovered that, in addition to NOX2-dependent production of oxygen radicals, murine macrophages have the capacity to produce nitrogen-based radicals that can contribute to microbial killing through an L-arginine-dependent biochemical pathway [231]. Such a study is proved to be more difficult to extrapolate to humans. Indeed, some studies showed low but detectable NO^·^ output [232, 233] that can be further increased by activating agents such as the chemotactic peptide N-formyl-L-methionyl-L-leucyl-L-phenylalanine [232]. Conversely, other studies failed to show NOS activity or the production of the NO^·^ metabolite nitrite and nitrate by human neutrophils, even after activation with proinflammatory cytokines [234, 235]. However, it is now indisputable that human macrophages produce NO^·^ and that NOS2, which is not expressed in macrophages at “rest,” is significantly enhanced in response to appropriate inflammatory stimuli [236]. The activity of this enzyme is required for the formation of nitrotyrosine around phagocytosed bacteria, most likely through the intermediate production of peroxynitrite [236].

## 16. Free Radicals as Signalling Molecules

According to several researchers, a new era of free radicals in biological systems began in the 1970s when Mittal and Murad provided evidence that hydroxyl radical induced the activation of guanylate cyclase and the genesis of the “second messenger” cyclic guanosine monophosphate (cGMP) [237]. However, as early as 1974, it had been reported that exogenously added hydrogen peroxide could mimic the signalling activity of insulin [238] and oxidation of key fat cell sulfhydryl's in response to insulin receptor interaction playing a role in mediating the glucose transport activation [239].

This revelation remained within small free radical research circles until, at the end of the 70s, Mukherjee and Lynn [240] described a NADPH oxidase in membranes isolated from fat cells, the activity of which was stimulated by preincubation of cells with insulin. This provided the basis for an attractive hypothesis for the mechanism of action of insulin, according to which the hormone, through the stimulated H_2_O_2_ production, would mediate membrane sulfhydryl oxidation and thus promote glucose transport. In fact, a few years later, insulin was shown to activate a plasma membrane enzyme system with the properties of a NADPH oxidase resulting in the downstream production of H_2_O_2_ [241], which plays a role in facilitating normal signal transduction by insulin.

Moreover, in the 1980s, publications reported that low concentrations of superoxide and hydrogen peroxide H_2_O_2_ could also stimulate cell proliferation in hamsters [242] and human [243] fibroblasts in culture. Since then, a large body of evidence has been accumulated that, at the cellular level, ROS, besides acting as a “second messenger” of the insulin and stimulating growth responses in a variety of mammalian cell types [244], also regulate a wide variety of physiological functions.

Indeed, ROS were found to play crucial roles in the activation of genes under the control of the transcription factor NF-*κ*B [245], be mediators in the biosynthesis of prostaglandins [246], function in embryonic development [247], and act as signalling molecules within the individual cell and among cells during their lifespan [248].

Evidence has been also obtained that ROS are involved in normal muscle contraction. This idea dates back to the 90s when Reid and collaborators [249] reported that low levels of ROS that are present in skeletal muscle under basal conditions are a requirement for normal movement and that antioxidant-mediated depletion of ROS from unfatigued skeletal muscle results in the inhibition of their contraction [250].

Furthermore, ROS contribute to complex functions, including blood pressure regulation [251] and cognitive function [252]. It has been also shown that, although an increased ROS formation rate has been postulated to be the major determinant of lifespan [253], several longevity-promoting interventions increase the generation of ROS that activate cellular stress pathways to dampen tissue degeneration to promote healthy aging [254]. This has suggested that living systems have not only adapted to a coexistence with free radicals but also developed various mechanisms for the advantageous use of free radicals in various physiological functions.

An important development in the field of the ROS beneficial effects was the discovery that, in organisms from simple bacteria to complex mammals, ROS can induce redox-sensitive signal cascades leading to increased expression of antioxidant enzymes.

For example, it was found that, following mouse [255] and rat [256] irradiation, cells and tissues appeared to respond by increasing the expression of cellular antioxidant defences, and this increased antioxidant capacity has been hypothesized to be at least partially responsible for radiation-induced adaptive responses. The increase in the effectiveness of the antioxidant defence system provided by this genetic response enables cells to survive an oxidant exposure that would normally be lethal.

Several studies showed that bacteria have evolved sophisticated molecular mechanisms to monitor oxidant levels and to activate antioxidant defence genes. In particular, the enteric bacterium *Escherichia coli* provided an excellent model to study gene regulation in response to oxidative stress. In fact, the basic principles of the oxidative stress response are universally conserved from bacteria to eukaryotes, although the exact mechanisms by which they sense ROS vary depending on the type and severity of stress condition and organism.

Thus, *E. coli* can stimulate the production of enzymes that scavenge the superoxide radical or various peroxides, of DNA repair enzymes, and of other proteins that mitigate the toxic effects of oxidative mutagens [257–259]. These protective inductions represent adaptive responses to oxidative stress because they are triggered by nontoxic levels of oxidizing agents or ionizing radiation and protect cells against a subsequent challenge with otherwise lethal levels of those oxidants.

The bacterial oxyR gene was identified in *Salmonella typhimurium* as a regulator of acquired resistance to oxidative stress, and it was found to be required for the expression of 9 of the many stress proteins, including catalase, alkyl hydroperoxide reductase, glutathione reductase, and MnSOD, synthesized in response to sublethal levels of hydrogen peroxide [260].

Subsequent researches defined a genetic locus, the soxR regulon [261, 262], which is part of the multigene response to superoxide generators and positively regulates 9 of the ~40 polypeptides distinct from those induced by H_2_O_2_ [263].

It was discovered that in mammal genes, transcription determining cell survival can be activated by ROS in two ways: either via transcription factors, which can interact directly with specific DNA motifs on promoters of target genes, or via activation of mitogen-activated protein kinase cascades, which in turn activate transcription factors that trigger target gene transcription [264].

Indeed, clear evidence was obtained that ROS cellular levels are strongly linked to the regulation of cellular antioxidant levels. One of the main examples of this effect is the control of the expression of several genes that codify for antioxidant and detoxifying enzymes by the nuclear factor erythroid 2-related factor 2 (Nrf2). Its action is mediated through the bond to the antioxidant response element (ARE) together with the small musculoaponeurotic fibrosarcoma (Maf) proteins, of the promoter region of the target genes [265]. The activity of Nrf2 depends on its subcellular localization that is, in turn, dependent, at least in part, by its bond with specific residues of cysteine of the protein Kelch ECH-associating protein 1 (Keap1) [266]. Furthermore, ROS can interact and activate several cellular processes among which the proliferation and survival through the interaction with signalling molecules such as mitogen-activated protein kinases (MAPKs), phosphoinositide 3-kinases (PI3K), phosphatase and tensing homolog (PTEN), and protein tyrosine phosphatases [267].

Similarly, the RNS also exert a dual role being harmful or beneficial to the living systems. Nitric oxide, which was initially discovered as a signalling molecule able to modulate in blood vessel diameter [121], was subsequently identified as a molecule able to induce cellular toxicity and damage metabolic enzymes and to generate, reacting with superoxide, the peroxynitrite [268], as well as a regulator of important physiological processes [269].

Nitric oxide is probably the smallest and most versatile bioactive molecule identified. Early studies concerning the role of NO^·^ in mammalian organisms showed that, besides inducing vasorelaxation, NO^·^ inhibited platelet aggregation [270]. However, it was soon evident that the effects and function of NO^·^ went beyond its ability to regulate vascular tone and cell adhesion.

For example, it was found that nitric oxide influences considerably the central and peripheral nervous systems. In fact, stimulation of cerebellar slices with excitatory amino acids led to the release of a labile mediator with pharmacologic properties like those of NO^·^, including the ability to raise cGMP in cerebellar cells that did not themselves respond to excitatory amino acids and the ability to relax vascular smooth muscle [271]. Subsequent studies on the peripheral nervous system provided the best evidence for a transmitter role of NO^·^. NOS inhibitors selectively blocked nonadrenergic noncholinergic- (NANC-) mediated relaxation of the gastrointestinal tract [272]. These data coupled with the selective localization of nNOS to the myenteric plexus indicated that NO^·^ functions as the NANC neurotransmitter [273].

Interestingly, the observation that lipid peroxidation can be inhibited by excess NO^·^ and yield a variety of nitroso- and nitro-fatty acid-derived products [274] also demonstrated that NO^·^ can also function as an antioxidant since it terminates free radical processes and stops radical chain propagation reactions.

## 17. Mechanisms of Redox Signalling

Even though the ROS and RNS were involved in both cellular damage and signal transduction was well established, several controversial questions remained open.

Differences in the concentrations of the ROS and RNS could, at least in part, be responsible for the dual role of such species as essential molecules for the regulation of cellular functions and as harmful by-products of metabolism. Indeed, at low concentrations, ROS played an important role as regulatory mediators in signalling processes, whereas, at moderate or high concentrations, they were harmful to cell organisms inactivating important cellular molecules. This was analogous to the effects of nitric oxide, which had both regulatory functions and cytotoxic effects depending on the enzymatic source and relative amount of NO^·^ generated [275]. Thus, NO^·^ worked as a signalling molecule mediating vasodilation when produced in low concentrations by the constitutive isoform of nitric oxide synthase (eNOS) in vascular endothelial cells [276]. Conversely, it worked as a source of highly toxic oxidants, such as peroxynitrite and nitrogen dioxide (NO_2_), utilized for microbicidal killing when produced in high concentrations by iNOS in macrophages [277].

Although these results suggested that the cellular levels of reactive species determine the shift from their beneficial to harmful effects, the concentrations to which this shift happens were and still remain generally unknown today.

Moreover, there was an apparent contrast between the specificity that is required for signalling and the general reactivity of cellular ROS that renders them indiscriminate and potentially lethal oxidants, so that it was unclear how any specificity in their opposite actions could be achieved. Specificity in signalling is achieved through the noncovalent binding of a ligand to its cognate receptor due to the complementarity of macromolecular shapes. By contrast, ROS operate in signalling through chemical reactions with specific atoms of target proteins that lead to covalent protein modifications. Therefore, ROS molecular recognition occurs at the atomic level and not at the macromolecular level, which necessarily expands the potential number of ROS-specific receptors. Thus, it was unclear how the specificity was achieved in ROS signalling.

The cell type, duration of oxidant production, reactive species produced, and localization of their source and their targets were suggested as contributing factors [216], but the information on this topic was still scarce and discordant.

.

### 17.1. ROS Source

Regarding the ROS source, mitochondria are considered the main source of ROS in the cell in both physiological and pathological conditions [278]. This is why mitochondria are considered to play the main role in many diseases and in the aging process [279]. In fact, the available data show that the evidence of mitochondria as the main source of ROS is lacking [280]. Conversely, they show that there is a strong interaction among the different sources of cellular ROS [281, 282], and this renders it more difficult to define the main source of reactive species in different physiological and pathological conditions.

### 17.2. Colocalization of Sources and Targets of ROS

Another primary layer of control for ROS signalling is the colocalization of sources and targets of ROS by the generation of the small-molecule oxidant in proximity to a given substrate. This form of regulation can directly influence the kinetics of a putative chemical signalling reaction by controlling the local concentrations of the participating molecular reactants. For example, NOX proteins that influence receptor tyrosine kinase signalling are often colocalized with their putative physiological targets, such as phosphatases and kinases, at the plasma membrane. This also prevents oxidation of pathological targets such as nucleotides that are confined to other parts of the cell [283, 284]. Indeed, recent data show that ROS generation is localized for signalling in various cell types [285]. Other examples of colocalization of ROS signalling sources and targets are ER localization of NOX4 and its target, the protein tyrosine phosphatase 1B (PTP1B) [286] and the localized generation of HOCl by myeloperoxidase in phagosomes for pathogen defence [287].

### 17.3. Species Involved in Signalling

Most of the ROS cannot diffuse far from the site of their production due to their high instability and reactivity and to the antioxidant cellular capacity. This is the case of the radical ^·^OH which possesses a half-life of about 10^−9^ s for its high reactivity, differently from H_2_O_2_ which has a half-life of about 1 ms [288]. This explains why H_2_O_2_ can diffuse far from its source, differently from ^·^OH which has a mean effective radius of action of about 30 A and reacts with or very near to the biomolecule that produced it. Thus, a hydroxyl radical formed in the mitochondria will be unlikely to have a direct effect on other parts of the cell, for example, DNA in the nucleus.

Moreover, ^·^OH has indiscriminate reactivity toward biological molecules, whereas other ROS, such as O_2_^·-^ and H_2_O_2_, each has preferred biological targets. Despite this, hydroxyl radical has also been thought to be involved in signalling by Mittal and Murad [237], who suggested that stimulation of guanylate cyclase by SOD was due to the formation of H_2_O_2_ which, reacting with superoxide, produced ^·^OH radicals that activated guanylate cyclase. This idea was refuted by Friebe et al. [289] who found that stimulation of guanylate cyclase by SOD was not influenced by ^·^OH and proposed that it was due to elimination of superoxide, thereby preventing its reaction with NO^·^. Thus, the idea is widely shared that ^·^OH radical cannot play any specific role in signalling.

This does not seem to apply also for the derivatives of the nitric oxide. It had been assumed that peroxynitrite has an impact on pathways, which, under physiological conditions, are regulated by tyrosine phosphorylation and dephosphorylation. Although peroxynitrite does not react directly with tyrosine, all secondary radicals arising from peroxynitrite promote protein tyrosine oxidation and nitration [290]. The tyrosine nitration blocks the respective signalling cascades so that the fact that NO_2_ irreversibly binds to proteins seemed to have a pathological impact on cellular function, rather than contributing to physiological intracellular signalling. This agreed with the idea that the biological impact of primary and secondary reactive species is different. Primary ROS and RNS are regulated by SOD, CAT, GPX, and NOS, respectively, and are predominantly associated with signalling having a specific physiological function for the regulation of intracellular signalling. Conversely, the secondary species are associated with oxidative stress. They should be evolutionarily developed for extracellular actions, predominantly as part of the innate immune system for killing bacteria. The intracellular release of such secondary species leads to deleterious consequences, as these are highly active, without a reliable control system for intracellular levels. It was, therefore, generally assumed that, in evolution, the primary species were developed for intracellular physiological signalling whereas the secondary species, which can cause damage to the cell, were developed for extracellular actions, such as the killing of bacteria. Nevertheless, publications are dealing with the signalling role of peroxynitrite [291].

### 17.4. H_2_O_2_ Signalling

Most of the papers on ROS-mediated intracellular signalling suggested either O_2_^·-^ or H_2_O_2_ as the major signalling molecules.

It was known that superoxide can oxidize thiols to thiyl radicals, which can initiate a chain reaction, but the rate constants for this reaction are rather low, about 10^3^ M^−1^ s^−1^ at pH 7.4 [292], which are insignificant in comparison to the rate constants (about 10^9^ M^−1^ s^−1^) for the cytosolic and mitochondrial superoxide dismutases [293]. When O_2_^·-^ is generated by the NADPH oxidase isoform, NOX2, on the outside of cells, such as endothelial ones, it can enter cells, resulting in signalling [294]. However, inside the cell, it is rapidly dismuted to H_2_O_2_ and O_2_ and targets that react with which have not been demonstrated to do so in vivo so that it is likely that superoxide acts as a precursor of H_2_O_2_.

H_2_O_2_ enzymatic production and degradation, which provide specificity for time and place, and its chemistry, which provides specificity for the oxidation of thiols, qualify the peroxide a suitable second messenger.

It has been proposed that H_2_O_2_ initiates cellular signalling through modifications of target protein molecules or changes of the intracellular redox state [295], even though the distinction between these mechanisms is not easy.

The main targets of H_2_O_2_ are thiol groups of cysteine residues, and the best-described modification of the protein molecules involves the oxidation of such residues. Oxidation of –SH groups results in the formation of several different products, including sulfenic acid derivatives (-SOH) [296], which can subsequently undergo further oxidation to sulfinic (-SO_2_H) and eventually sulfonic (-SO_3_H) acid. Furthermore, they can form disulphide bonds with nearby cysteines (-S-S-) [297] and be transformed into several adducts by reaction with NO^·^ (S-nitrosylation) or GSH (S-glutathiolation) [298]. Except for sulfonic acid and to a lesser degree sulfinic acid, the modifications are reversible by reducing systems such as thioredoxin and peroxiredoxins [299].

The sulfinic acid formation requires a second reaction with H_2_O_2_ which seems more likely to occur under severe oxidative stress than during physiological signalling. The same is true for the sulfonic that practically does not exist in biological conditions. It follows that the oxidation states of a cysteine residue relevant for signalling are likely only the sulfenic acid derivative and the disulphide.

The oxidation-linked modifications of thiol groups result in changes in the structure and function of the protein, which may modify the activity of an enzyme if the critical cysteine is located within its catalytic domain [300] or the ability of a transcription factor to bind DNA if it is located within its DNA-binding motif [301]. Several proteins, including transcription factors, molecular chaperones, and protein tyrosine phosphatases, are regulated via redox processes. The redox state of protein thiols represents a “molecular switch” able to reversibly activate/deactivate protein function. This process resembles the phosphorylative regulation in which the addition of a phosphate group by a protein kinase and its removal by a protein phosphatase reversibly change protein activity [302].

On the other hand, crosstalk and/or sequential partnership between the two signalling mechanisms occur in some cases of cell regulation. Evidence linking the cellular redox state to a phosphorylative process came from the study of the Trx/ASK1 system [303]. Apoptosis signal-regulating kinase-1 (ASK1) is a member of the family of mitogen-activated protein kinase kinase kinases (MAPKKK) that is involved in the activation of stress-activated protein kinases, such as p38 and JNK [304]. Thioredoxin, an antioxidant protein, was found to form a complex with ASK1. When thioredoxin was complexed to ASK1, the activity of ASK1 was inhibited [303]. The rise in ROS levels that occurred following tumour necrosis factor stimulation resulted in the dissociation of ASK1 from thioredoxin and the subsequent activation of ASK1 activity [303].

As for how the changes in the redox cellular state initiate cellular signalling should be noted that, in comparison with the extracellular environment, the cytosol is normally maintained under strong “reducing” conditions. In normal conditions, the maintenance of the cytosol redox state is due to the “redox-buffering” capacity of intracellular thiols, such as GSH and Trx, which oppose cellular oxidative stress by reducing H_2_O_2_. The activity of GSH reductase and Trx reductase maintains the high ratios of reduction to oxidized forms of GSH and Trx, respectively. Such substances can participate in cell signalling processes: GSH can regulate redox signalling by alterations both in the level of total GSH and in the ratio between its reduced and oxidized forms, while Trx can regulate the activity of some proteins by directly binding to them [295].

### 17.5. NO Signalling

The well-known effect of NO^·^ on smooth muscle cells is due to the capacity of nitric oxide to bind to the heme moiety of guanylyl cyclase to induce a conformational change which activates the enzymes and results in the formation of the second messenger cyclic guanosine monophosphate (cGMP) from guanosine 5′-triphosphate (GTP). cGMP in turn, regulates protein kinases (cGK), phosphodiesterases (PDE), and ion-gated channels. Since the initial elucidation of the effects of the ^·^NO/cGMP system on blood pressure regulation, platelet aggregation, and neurotransmission [305], other examples of the alternative target for NO^·^ came to light. Some examples are the activation of another heme-containing enzyme, cyclooxygenase, which leads to an increase in prostaglandin formation [306], and the inhibition of cytochrome oxidase, which regulates mitochondrial respiration [307].

Many biological effects of NO^·^ are mediated through heme-independent pathways by which S-nitrosylation of protein-associated targets of NO includes cysteine and tyrosine residues, and it is apparent that S-nitrosylation of target proteins is another potentially important regulatory system accounting for some of NO^·^ physiologic actions.

It was found that eNOS and iNOS are susceptible to NO-induced thiol modifications [308]. S-nitrosylation of NOS enzymes regulates the NO^·^ production suggesting a feedback mechanism to control its activity [308]. NO^·^ also targets the mitochondrial proteins complex I and complex IV involved in respiration. Although the inhibition of complex IV remains reversible throughout the time of exposure, the inhibition of complex I, which seems to be due to S-nitrosylation of this enzyme, becomes progressively persistent as the concentration of reduced glutathione (GSH) in the cell decreases [309].

Like NO^·^, peroxynitrite easily crosses biological membranes, and despite a relatively short half-life (~10 ms), it can interact with target molecules in adjacent cells within one or two cell diameters [269]. Peroxynitrite can indirectly trigger the nitration of tyrosine residues in proteins, forming 3-nitrotyrosine, via the generation of highly reactive radicals formed by its reaction with carbon dioxide [310].

Protein tyrosine nitration is a covalent modification resulting from the addition of a nitro (-NO_2_) group onto one of the two equivalent ortho carbons of the aromatic ring of tyrosine [310]. The nitration may produce three distinct effects on the affected proteins: loss of function, the gain of function, or no effect [311]. Widespread tyrosine nitration occurred in cells during *in vitro* exposure to peroxynitrite, affecting structural proteins, ion channels, metabolic enzymes, and proteins involved in apoptosis, to name only a few [311–313]. The relevance of such observations *in vivo* remained, however, to be established, given that the yield of nitrotyrosine formation under conditions of elevated peroxynitrite generation *in vivo* (nitrooxidative stress) remained largely smaller than what can be achieved *in vitro* with direct peroxynitrite exposure.

However, in most reported studies, nitration of tyrosine has been associated with a significant loss of function of the nitrated protein. An important example of the loss of enzyme activity is that of mitochondrial MnSOD, the first protein to be found nitrated in vivo [314]. Nitration of a single tyrosine residue (Tyr-34) leads to complete enzyme inactivation, with the possible consequence of favouring peroxynitrite generation in this organelle, due to the impaired dismutation of O_2_^·^ [315].

A major aspect of tyrosine nitration by peroxynitrite is the possibility of impairment of cellular processes depending on the generation of phosphotyrosine [269]. It is worth noting that the hypothesis that tyrosine nitration would essentially inhibit phosphotyrosine-dependent cell signalling has been largely reviewed following a series of investigations concluding that peroxynitrite rather promoted the process of tyrosine phosphorylation in a variety of cell types [291]. Proposed mechanisms involve an imbalance between tyrosine phosphorylation and dephosphorylation, related to the activation of tyrosine kinases or the inhibition of tyrosine phosphatases, respectively, by peroxynitrite. However, the scarcity of *in vivo* results that confirm *in vitro* data indicates that the relevance of peroxynitrite-dependent signalling in pathological states remains to be specified.

## 18. Stress Antioxidative

The aforementioned results indicate that metabolic pathways of aerobic organisms lead to the obligate production of reactive oxygen species (ROS) and reactive nitrogen species (RNS), which are not necessarily bad. Reactive species are actually needed as signal transduction elements in processes that are essential for life.

Just as reactive species are not necessarily bad, antioxidants are not necessarily good. It is well accepted that oxidative stress is produced when the equilibrium between reactive species and antioxidants is tilted in favour of the former. However, it is now well established that the equilibrium can be broken also if antioxidant levels exceed those of the reactive species, and Dündar and Aslan [316] proposed the term “antioxidative stress” for such disequilibrium.

First, antioxidants play the role of blocking free radical production and oxidative stress, but they are not able to distinguish among radicals which produce damage and those that play a physiological role. Moreover, these compounds not only function as antioxidants but also have prooxidant action. For example, vitamin C is a well-known antioxidant, and Linus Pauling reported a pronounced effect of vitamin C in decreasing the incidence and delaying the onset of malignant lesions in rats [317] and mice [318] exposed to ultraviolet light. However, by now, a vast number of nutritional intervention trials using vitamin C, as well as other antioxidants, have shown no efficacy in preventing gastrointestinal cancer and delaying death [319]. Rather, they seem, to slightly shorten the lives of those who take them.

On the other hand, there is evidence that ascorbic acid can also act as a prooxidant. Vitamin C does not affect itself, but the combination with iron causes intense oxidation of polyunsaturated fats.

The superoxide ion is not a particularly reactive radical, and its toxicity is linked to the fact that it can reduce the Fe^3+^ ion to Fe^2+^ ion restoring a species, H_2_O_2_, which can produce the radical ^·^OH. Ascorbic acid also converts Fe^3+^ to Fe^2+^ thus allowing another cycle of ^·^OH generation from the renewed ferrous ion [320]. Integration with ascorbic acid leads to a constant supply of reduced ascorbic acid, thus leading to a repetitive generation of ^·^OH radicals by iron [320, 321]. Haematologists who studied iron overload due to transfusion therapy of patients with thalassemia or sickle cell disease reported that vitamin C can mobilize iron from their body iron stores to overwhelm the iron-binding capacity of iron-binding proteins and the resultant free iron-producing death within minutes to hours from iron-induced cardiac failure [322, 323]. Thus, vitamin C supplements can cause rapid progression to death of the otherwise more slowly progressive congestive cardiomyopathy of haemochromatosis [324]. Three young athletes so died, and no one thought of assessing their iron status before letting them take vitamin C supplements [325].

Moreover, because at low doses ROS possess a crucial role in many physiological functions, the balance between oxidant production and antioxidant protection is believed to be critical in maintaining healthy biological systems. Therefore, antioxidants at high doses besides acting as prooxidants, could also disrupt the redox balance following their potential to interact with ROS present at physiological concentrations required for optimal cellular functioning, leading to cellular dysfunction [326].

Furthermore, antioxidant supplementation can inhibit the adaptive response to ROS. A paradigmatic example is provided just by exercise. Indeed, a single session of strenuous or prolonged exercise leads to the production of a high number of radicals and other reactive oxygen species (ROS), which cause tissue damage and dysfunction. Conversely, the single sessions of a training program produce low amounts of ROS which can induce adaptive responses beneficial for the organism [327].

Interestingly, regular exercise appears to decrease the incidence of a wide range of ROS-associated diseases, including heart disease, T2DM, rheumatic arthritis, Alzheimer's and Parkinson's diseases, and certain cancers [328, 329].

The main adaptations occur in the trained skeletal muscle which can differ with the type of exercise but seem to be nevertheless dependent on ROS production. Thus, aerobic physical activity induces skeletal muscle adaptive responses [328], which increase resistance to conditions leading to increased ROS production including prolonged or strenuous exercise [330–332]. Conversely, heavy resistance exercise results in hypertrophy of the muscle cells with an increase in strength, without major changes in biochemical makeup.

An important concept developed over the past decade is that the responses to training are likely the result of the acute but cumulative effects of the responses to single exercise bouts [333]. Thus, each bout of exercise initiates acute and transient changes in gene transcription which are reinforced by repeated exercise stimuli, leading to altered, chronic expression of a variety of nuclear and mitochondrial DNA (mtDNA) gene products, which ultimately form the basis of skeletal muscle training adaptation and improvements in exercise capacity [334]. Training also slows down peroxidative processes [331, 335] and the appearance of other signs of free radical generation [331] induced by acute exercise in rat skeletal muscle. This effect is associated with increased antioxidant defences [331, 336] and decreased free radical activity [337, 338].

It was suggested that the ROS generated during the single exercise sessions act as signals regulating molecular events crucial for the adaptive responses to training. It was also proposed that antioxidant supplementation, decreasing ROS formation, prevents useful adaptations for muscular cells [339]. Such an idea was tested determining the effects of the antioxidant supplementation on exercise-induced adaptive responses. Investigations concerning the effects of higher intakes of vitamin C and/or E on exercise performance and redox homeostasis have supplied contrasting results [340]. However, antioxidant supplementation has been more consistently reported to prevent health-promoting effects of physical exercise in humans [341, 342].

## 19. Conclusions

Our excursion into the field of free radicals has led us to point out that the scientific community has had to revise its ideas around these substances in quite a short time. Initially seen as substances that cannot be isolated due to the limitations of the available methods, they were then considered as short-lived substances, but important for their involvement in the combustion and organic synthesis processes. Subsequently, they were considered biochemical intermediates naturally occurring in biological systems, but impossible to identify. Thanks to the technique of electron spin resonance it was shown that free radicals were much more widespread in biological systems than previously assumed. The next step was the recognition that these species were responsible for the toxicity of oxygen and the deleterious effect of ionizing radiation.

Then came the evidence that free radicals were by-products of normal cellular metabolism and that their harmful effects were counteracted by an antioxidant defence system.

When the balance between free radical production and the capacity of antioxidant systems to neutralize them was disturbed in favour of the former, oxidative stress arose. This led to oxidative modification of biological macromolecules with consequent cell structural and functional dysfunction leading to diseases and aging.

The most recent stage has been the recognition that radicals at low concentrations can perform important functions in biological systems and that antioxidants can prevent such actions.

If subsequent researches will confirm such results, they will lead to a definitive rethinking of the idea that there has been for a long time about radicals and antioxidants. Above all, the still widespread habit of considering such substances as good and bad, respectively, would fall. Conversely, the idea would be affirmed that the goodness and badness of these substances are relative and are dependent on a series of factors, among which their concentration is very important.

## Figures and Tables

**Table 1:** Steps in evolving knowledge of free radicals.

Toxic effects of oxygen on central nervous system (Paul Bert effect)	Bert [61]
Pulmonary toxicity of oxygen (Lorrain Smith effect)	Smith [62]
Preparation of the triphenylmethyl radical, (C_6_H_5_)_3_C^·^	Gomberg [5]
Properties of xanthine oxidoreductase (XOR)	Schardinger [100]
Oxygen effect on radiosensitivity	Schwartz [44]
Isolation of the atomic hydrogen	Wood [11]
Preparation of the free radical methyl (^·^CH_3_)	Paneth and Hofeditz [13]
“Activated solvent” hypothesis for indirect action of ionizing radiation	Risse [40]
Discovery of free radicals as biochemical intermediates in biological systems	Michaelis [29]
First utilization of X-rays for cancer treatment	Grubbe [218]
Discovery of the “peroxide effect”	Kharasch and Mayo [14]
Generation of the hydroxyl radical	Haber and Weiss [27]
Suggestion of a link between retinopathy and excess of oxygen	Campbell [64]
Involvement of free radical in oxygen toxicity	Gerschman et al. [68]
Observation of free radicals in biological systems by ESR	Commoner et al. [69]
Detection by ESR of a semiquinone during the riboflavin oxide-reduction	Beinert [31]
Implication of free radicals in biological aging	Harman [70]
Formation of H_2_O_2_ by microsomal NADPH oxidase	Gillette et al. [86]
Spin restriction in oxygen reactivity	Taube [67]
Discovery of the superoxide dismutase (SOD)	McCord and Fridovich [71]
“Superoxide theory” of oxygen toxicity	McCord et al. [74]
Generation of H_2_O_2_ by pigeon heart mitochondria	Loschen et al. [82]
Mitochondrial formation of H_2_O_2_ under hyperbaric conditions	Boveris and Chance [85]
Superoxide as initial product of respiratory burst	Babior et al. [95]
H_2_O_2_ mimics the signaling activity of insulin	Czech et al. [238]
Stimulation of NADPH oxidase by insulin	Mukherjee and Lynn [240]
Activation by ^·^OH radical of guanylate cyclase	Mittal and Murad [237]
Oxygen radical involvement in reperfusion injury	Granger et al. [102]
Observation by ESR of ROS production during exercise	Davies et al. [199]
Formation of peroxynitrite from nitric oxide and superoxide	Blough and Zafiriou [129]
Definition of “oxidative stress”	Sies [157]
Increase in lipid peroxidation in hyperthyroid rat liver	Fernández et al. [181]
Identification of bacterial oxyR gene	Christman et al. [260]
Identification of endothelial-derived relaxing factor (EDRF) in NO^·^	Ignarro et al. [121]; Khan and Furchgott [122]; Palmer et al. [123]
Purification of nitric oxide synthase (NOS)	Bredt and Snyder [125]
Relationship between free radicals and muscle fatigue	Reid et al. [249]
Training slows down peroxidative processes during acute exercise	Venditti and Di Meo [331]
Discovery of Nrf2	Itoh et al. [265]
Training decreased free radical activity	Venditti et al. [337]
Mechanisms by which ROS initiate cellular signaling	Thannickal and Fanburg [295]
Antioxidant supplementation prevents training-induced useful adaptations for muscular cells	Gomez-Cabrera et al. [339]
ROS generation promotes healthy aging	Ristow and Schmeisser [254]

**Table 2:** Researchers in the field of free radicals and antioxidants awarded with the Nobel Prize.

Fritz Haber	1918 Chemistry	For the ammonia synthesis process
Albert Szent-Györgyi	1937 Medicine	For his discoveries on biological combustion processes, with particular reference to vitamin C and fumaric acid catalysis
Linus C. Pauling	1954 Chemistry	For his researches in the field of the molecular attraction and its applications for the explanation of the structure of complex substances
Nikolaj N. Semënov	1956 Chemistry	For his researches on mechanisms of chemical reactions
Cyril N Hinshelwood	1956 Chemistry	For his researches on mechanisms of chemical reactions
Gerhard Herzberg	1971 Chemistry	For his contributions to the knowledge of electronic structure and the geometry of molecules, in particular free radicals
Louis J Ignarro	1998 Medicine	For the discovery of NO^·^ as signal molecule
Ferid Murad	1998 Medicine	For the discovery of NO^·^ as signal molecule
Robert F. Furchgott	1998 Medicine	For the discovery of NO^·^ as signal molecule

**Table 3:** Selected diseases/disorders for which a role of free radicals has been suggested.

Adult respiratory distress syndrome	Gout
Allergic encephalomyelitis	Haemachromatosis
Alzheimer's disease	Hearing loss
Amyotrophic lateral sclerosis	Hypertension
Asbestosis	Insulin resistance
Autism	Keshan disease
Autoimmune vasculitis	Lypofuscinosis
Bloom syndrome	Malaria
Bronchopulmonary dysplasia	Multiple sclerosis
Burns	Muscular dystrophy
Cancer	Myasthenia gravis
Cataract	Pancreatitis
Chronic autoimmune gastritis	Parkinson disease
Chronic granulomatous disease	Psoriasis
Cirrhosis	Psychosis
Contact dermatitis	Retinal degeneration
Depression	Retrolental fibroplasias
Dermamyositis	Rheumatoid arthritis
Dermatomyositis	Schizophrenia
Diabetes mellitus	Sickle cell anemia
Emphysema	Stroke
Emphysema	Systemic lupus erythematosus
Favism	Thalassemia
Glomerulonephritis	Ulcerative colitis

## Supplementary Materials

Supplementary Materials The figure is a graphical abstract.
