# Supplementary figures and images for: Evolution of the Knowledge of Free Radicals and Other Oxidants
Source: Oxid Med Cell Longev. 2020 Apr 23;2020:9829176. doi: 10.1155/2020/9829176 (PMC7201853; doi:10.1155/2020/9829176)

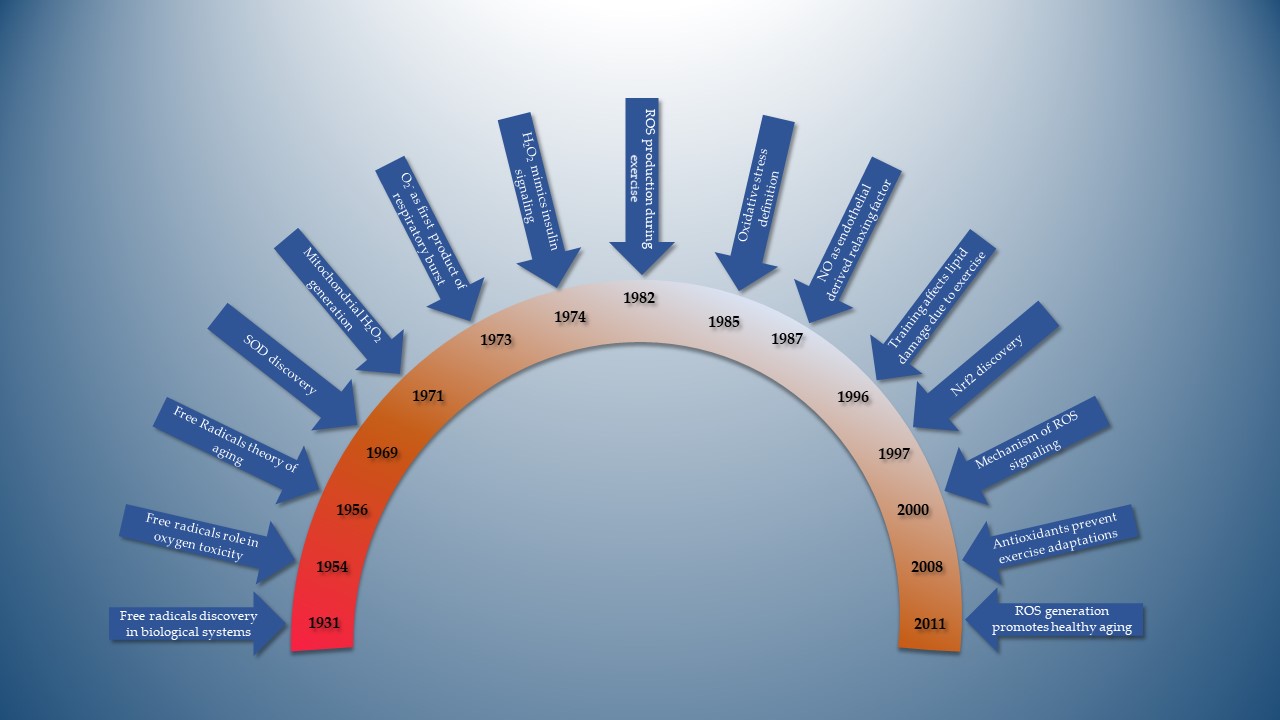

Supplement: Supplementary Materials — The figure is a graphical abstract. [file 9829176.f1.jpg]
